# M2 macrophage-derived exosomes promote the c-KIT phenotype of vascular smooth muscle cells during vascular tissue repair after intravascular stent implantation

**DOI:** 10.7150/thno.46143

**Published:** 2020-08-29

**Authors:** Wenhua Yan, Tianhan Li, Tieying Yin, Zhengjun Hou, Kai Qu, Nan Wang, Colm Durkan, Lingqing Dong, Juhui Qiu, Hans Gregersen, Guixue Wang

**Affiliations:** 1 Key Laboratory of Bio-Rheological Science and Technology, State and Local Joint Engineering Laboratory for Vascular Implants, Bioengineering College of Chongqing University, Chongqing, China.; 2 GIOME, Department of Surgery, the Chinese University of Hong Kong, Hong Kong, China.; 3 The Nanoscience Centre, University of Cambridge, Cambridge, UK.; 4 Cavendish Laboratory, University of Cambridge, Cambridge, UK.

**Keywords:** M2 macrophage, exosomes, stiffness, vascular smooth muscle cell, dedifferentiation, intravascular stent

## Abstract

**Rationale:** For intravascular stent implantation to be successful, the processes of vascular tissue repair and therapy are considered to be critical. However, the mechanisms underlying the eventual fate of vascular smooth muscle cells (VSMCs) during vascular tissue repair remains elusive. In this study, we hypothesized that M2 macrophage-derived exosomes to mediate cell-to-cell crosstalk and induce dedifferentiation phenotypes in VSMCs.

**Methods:** *In vivo*, 316L bare metal stents (BMS) were implanted from the left iliac artery into the abdominal aorta of 12-week-old male Sprague-Dawley (SD) rats for 7 and 28 days. Hematoxylin and eosin (HE) were used to stain the neointimal lesions. *En-face* immunofluorescence staining of smooth muscle 22 alpha (SM22α) and CD68 showed the rat aorta smooth muscle cells (RASMCs) and macrophages. Immunohistochemical staining of total galactose-specific lectin 3 (MAC-2) and total chitinase 3-like 3 (YM-1) showed the total macrophages and M2 macrophages. *In vitro*, exosomes derived from IL-4+IL-13-treated macrophages (M2Es) were isolated by ultracentrifugation and characterized based on their specific morphology. Ki-67 staining was conducted to assess the effects of the M2Es on the proliferation of RASMCs. An atomic force microscope (AFM) was used to detect the stiffness of the VSMCs. GW4869 was used to inhibit exosome release. RNA-seq was performed to determine the mRNA profiles of the RASMCs and M2Es-treated RASMCs. Quantitative real-time PCR (qRT-PCR) analysis was conducted to detect the expression levels of the mRNAs. Western blotting was used to detect the candidate protein expression levels. T-5224 was used to inhibit the DNA binding activity of AP-1 in RASMCs.

**Results:** M2Es promote c-KIT expression and softening of nearby VSMCs, hence accelerating the vascular tissue repair process. VSMCs co-cultured *in vitro* with M2 macrophages presented an increased capacity for de-differentiation and softening, which was exosome dependent. In addition, the isolated M2Es helped to promote VSMC dedifferentiation and softening. Furthermore, the M2Es enhanced vascular tissue repair potency by upregulation of VSMCs c-KIT expression via activation of the c-Jun/activator protein 1 (AP-1) signaling pathway**.**

**Conclusions:** The findings of this study emphasize the prominent role of M2Es during VSMC dedifferentiation and vascular tissue repair via activation of the c-Jun/AP-1 signaling pathway, which has a profound impact on the therapeutic strategies of coronary stenting techniques.

## Introduction

Coronary stenting has almost universally superseded the use of balloon angioplasty alone for the percutaneous treatment of coronary disease 1. However, vascular injury caused by stent implantation always leads to restenosis because of the uncontrollable proliferation of vascular smooth muscle cells (VSMCs) 2. The proliferation of VSMCs in the vascular injury area can be caused by many factors, but crucial among them is the stimulation of infiltrated macrophages 3,4. Macrophages, including M1 and M2 macrophages, participate in the repair of vascular injury after stent implantation 5,6. However, the mechanism underlying the effects of macrophages on the fate regulation of VSMCs during vascular tissue repair and therapy remains elusive.

Recently, extracellular vesicles (EVs) have been considered an orchestrator between different kinds of cells, in local or distant microenvironments and within multiple pathophysiological processes. For example, EVs from macrophage foam cells can promote VSMC migration and adhesion, which might be mediated by the integration of EVs into VSMCs 4. Exosomes from nicotine-treated macrophages accelerate the development of atherosclerosis by increasing VSMC migration and proliferation through its target PTEN 7. Exosomes derived from M1 macrophages have been shown to aggravate neointimal hyperplasia by delivering miR-222 into VSMCs 8. Nonetheless, there is still no definitive conclusion as to whether or how M2 macrophage exosomes affect the behavior of VSMCs during vascular tissue repair after stent implantation.

On the other hand, VSMCs may exhibit stem cell properties, including the expression of stem cell markers (such as c-KIT) 9, softness 10, and high proliferation in response to inflammation or other microenvironmental factors in the vascular injury site. Stem cell marker expression by VSMCs occurs in the early injury phases, which probably plays a major role in vascular remodeling 11,12. Although it is well accepted that c-KIT^+^ VSMCs can modulate vascular remodeling, the detailed mechanisms by which inflammatory cells regulate VSMC functions and the related mechanism remain unclear.

The objective of this work was to explore the mechanism of cell conversion in the stented area, as well as to understand the significance of conversion to neointimal formation. This work shows that most of the c-KIT^+^ VSMCs around the stent struts originate from the medial VSMCs. M2 macrophage exosomes (M2Es) specifically regulate VSMC dedifferentiation by activating c-Jun/activator protein 1 (AP-1) around the stent struts, enhancing the vascular repair potency.

## Methods

### Stent implantation in rat aortas

The detailed methods are available in the Online Appendix. In brief, 12-week-old male Sprague-Dawley (SD) rats were pre-dosed orally with aspirin and clopidogrel 3 days before surgery, and they were maintained on this dual antiplatelet therapy throughout the study to reduce the risk of in-stent thrombosis. Vascular access was obtained by left femoral artery cutdown and the insertion of a homemade sheath. A stainless steel stent (8-cell, 2.5 mm × 0.8 mm; Amsino Medical, Beijing, China) was pre-mounted on VasoTech^®^ Miniature balloon catheters (15 mm × 1.5 mm, VasoTech, Inc.) and deployed (10 ATM for 30 s, 3 times) into the abdominal aortic. The rats were allowed to recover in heated chambers for 24 h and were then returned to normal housing conditions, where they were maintained on aspirin + clopidogrel-supplemented food for another 7 or 28 days before being sacrificed.

To deliver the M2Es into the stented abdominal aorta and to avoid any potential systemic side effects, we applied an established local delivery model via the pluronic gel F-127, as described in previous reports with minor modification 13,14. Briefly, immediately after stent implantation, 50 µg M2Es in phosphate buffer saline (PBS) preloaded into 50 µL 20% pluronic gel F-127 (Sigma) at 4 °C was applied locally to the adventitia around the injured artery segments. Then, M2Es (10 µg) in PBS or vehicle (PBS) were injected into the tail intravenously every three days until the rats were sacrificed.

### Electrolysis

Following fixation in 4% PFA, the stented murine arteries were immersed in 5% (w/v) citric saline, and electrochemical dissolution was performed by passing a small direct current through the stent as described previously 15.

### H&E staining

Hematoxylin and eosin (H&E) staining was performed after the rats were euthanized and the stents were electrolyzed. Artery samples were fixed in 4% PFA for 24 h, and then paraffin-embedded. They were sectioned (5 µm sections) and stained with H&E. An optical microscope (Leica, DMI6000B, Germany) was used to analyze these slides.

### Cell culture

A line of rat VSMCs (A7R5, ATCC) was cultured in high-glucose Dulbecco's modified Eagle medium (DMEM, Gibco) with 10% fetal bovine serum (FBS, Gibco). THP-1 human acute monocytic leukemia cells were kindly provided by the Stem Cell Bank, Chinese Academy of Sciences. Cells were cultured in RPMI-1640 containing 10% FBS (Gibco BRL). Cells were maintained at 37 °C in a 5% CO_2_ humidified environment. To mimic a pro-inflammatory environment and consequently promote M1 or M2 polarization, the M1 cells were stimulated with lipopolysaccharide (LPS) and interferon-γ (IFN-γ). For M2 polarization, the cells were stimulated with interleukin-4 (IL-4) and interleukin-13 (IL-13) were added back with DMSO (carrier) for 48 h 16.

Rat aorta smooth muscle cells (RASMCs) were isolated from the thoracic aorta of male SD rats (200-250 g body weight) using the explant technique. Cells were cultured in DMEM supplemented with 20% FBS and 1% penicillin/streptomycin and incubated in 5% CO_2_ at 37 °C. Primary RASMCs from passages 5 to 12 at 80% confluence were used for the experiments. The cells were serum-starved (5 mM glucose with 0.2% bovine serum albumin (BSA)) for 24 h before use in the experiments. T-5224 (10 µM, HY-12270, MCE) was used to inhibit the DNA binding activity of AP-1 in the RASMCs.

### Exosome purification and characterization

For M2 macrophage exosome isolation, the cells were incubated with 10µM DID (1,1'-dioctadecyl-3,3,3',3'-tetramethylindodicarbocyanine perchlorate) for 20 min, and afterwards washed 3 times with PBS for 5 min each time. Then, the cells were cultured in exosome depleted medium for 48 h before supernatant collection. The extracellular medium was concentrated using a Centricon Plus-70 centrifugal filter. Debris and dead cells in the medium were removed by centrifugation at 1000 × g for 10 min at 4 °C, and subsequently the supernatants were transferred to a fresh tube and centrifuged at 10,000 g for 30 min at 4 °C. The medium was then subjected to ultracentrifugation at 100,000 g for 70 min at 4 °C. After washing with PBS (100,000 g for 70 min), the exosome containing pellet was re-suspended in PBS. Nanoparticle Tracking Analysis (NTA) particle size characterization of the exosomes was carried out by NanoSight analysis (Malvern Instruments).

### RNA-seq and Real-time RT-PCR

The quality of the RNA for sequencing was determined using a Bioptic Qsep100 Bioanalyzer. Library preparation used the KAPA RNA-Seq Library Preparation Kit (KAPA Biosystems Cat#07960140001) and the RNAs were single-end sequenced on Illumina HiSeq 3000 machines. The reads were aligned using HISAT2 with the default parameters for the rat genome. Transcript assembly and differential expression were determined using Cufflinks with Refseq mRNAs to guide assembly. RNA-seq data were analyzed using the cummeRbund package in R. The heatmap was generated with Heatmap Builder. Gene set enrichment analysis was performed using the GSEA app (Broad Institute) based on the Kyoto Encyclopedia of Genes and Genomes (KEGG) gene lists.

Briefly, RNAs from VSMCs were isolated with an RNA isolation kit (Takara, Inc.). qRT-PCR for *SM22α*, *α-SMA*, *c-Kit*, *Oct-4*, *Klf4*, *Nanog*,* c-Myc*,* Sox2*,* Kitlg*,* Map2k1*,* Fosl1*,* Opn*,* Elastin*,* Mgp*,* Jun* and *Sca-1* was performed on cDNA generated from 1000 ng of total RNA using the protocol of the qRT-PCR mRNA Detection Kit (Takara). Amplification and detection of the specific products were performed with a Roche Lightcycler 480 Detection System. As an internal control, *Gapdh* was used for the other template normalizations. Fluorescent signals were normalized to an internal reference, and the threshold cycle (Ct) was set within the exponential phase of the PCR. The relative gene expression was calculated by comparing cycle times for each PCR target. The target PCR Ct values were normalized by subtracting the *Gapdh* Ct value, which generated the ΔCt value. The relative expression level between treatments was then calculated using the following equation: relative gene expression = 2 ^-(ΔCtsample-ΔCtcontrol)^.

### Western blotting

The method used was similar to that described previously 16. Cells were harvested and washed with cold PBS and then re-suspended in lysis buffer (Beyotime, China) supplemented with protease inhibitors (Roche). The protein concentration was determined using the Bio-Rad Protein Assay Reagent and 50 µg of whole lysate was applied to SDS-polyacrylamide gel electrophoresis (PAGE). They were transferred to Hybond polyvinylidene fluoride (PVDF) membranes (Millipore Immobilon-P), followed by standard western blot procedures. After blocking with 5% skimmed milk powder in Tris-buffered saline Tween-20 (TBST), the membranes were incubated with each of the following antibodies at 4 °C for 18 h: SM22α (Abcam, #ab14106), α-SMA (Abcam, #ab21027), c-KIT (Santa Cruz, #sc-365504) and GAPDH (CST, #5174). The bound primary antibodies were detected by the use of an HRP-conjugated secondary antibody and an ECL detection system (CST). The band density was semiquantified by ImageJ2x.

### AFM

For Atomic Force Microscope (AFM) characterization of the cells, a Nanowizard II AFM (JPK Instruments AG, Berlin, Germany), as described previously, was used. For a control group, VSMC were cultured in serum-free DMEM for 24 h. We divided the experimental group into three subgroups, RASMC treated with M2 Macrophage supernatant, RASMC co-cultured with M2 macrophages in a Transwell (membrane pore =0.4 µm) plate and RASMC treated with M2Es for 24 h, respectively. Imaging of the washed VSMC was done in contact mode in air and under PBS with silicon and V-shaped silicon nitride probes (SICON-Applied Nanostructures and PNP-TR-Nanoword). The resultant spring constant was evaluated as 0.01 N m^-1^. Force spectroscopy on the living cells was conducted with V-shaped soft silicon nitride probes (MLCT. Bruker). The force curves were analyzed by Atomic J to calculate the sample Young's modulus using the Hertz model 17. AFM image processing of the samples was done using the JPK data processing software (v.4.4.29). Each group analyzed at least ten cells, and for each cell at least twenty points were measured. The stiffness of each cell was calculated from the average stiffness of all of the effective points on each cell.

### TEM

For transmission electron microscopy (TEM), exosomes were fixed with 2.5% glutaraldehyde and loaded on formvar and carbon-coated copper grids. The grids were placed on 2% gelatin at 37 °C for 20 min, rinsed with 0.15 M glycine/PBS, and the sections were blocked using 1% cold water fish-skin gelatin. Grids were viewed using a JEOL 1200EX II (JEOL) transmission electron microscope and photographed using a Gatan digital camera (Gatan).

### Co-culture assay

After transfection with DII (1,1-dioctadecyl-3,3,3,3-tetramethylindocarbocyanine iodide) (10 µM) for 20 min, they were washed 3 times with PBS for 5 min each time. Macrophages (0.1-3×10^5^/well) were co-cultured with A7R5 cells (incubated with 10 µM DIO (3,3′-dioctadecyloxacarbocyanine perchlorate)) or RASMCs at a ratio of 1:1 using a Transwell plate (0.4 µm polycarbonate filter, Corning) for 24 h, with A7R5 cells or RASMCs placed in the lower chamber and macrophages in the upper chamber. After washing with PBS three times, the appearance of DII red fluorescence on the A7R5 or RASMCs was examined. GW4869 (Sigma-Aldrich, 10 µM) was added to block exosome secretion.

### Immunofluorescence staining

In brief, vessels were fixed in 4% paraformaldehyde. Rat stented aortas were electrolyzed to remove the stents, as described above, dehydrated in graded ethanol, cleared in Histo-Clear and embedded in paraffin wax, before sectioning and subsequent staining. Serial paraffin sections were dewaxed and rehydrated. Tissue sections were subjected to citric acid antigen retrieval, and endogenous peroxidase activity was inhibited by incubation with 3% hydrogen peroxide. Sections were incubated with CD68 antibody (Abcam, ab955, 1:200), c-KIT antibody (Santa Cruz, sc-365504, 1:100) or SM22α antibody (Abcam, ab14106, 1:200) overnight at 4 °C after blocking with 1% donkey or goat serum, followed by incubating with secondary antibody at room temperature for 1 h away from the light. Images were observed with a laser scanning confocal microscope (Lecia, SP8, Germany).

For cell immunofluorescence, cells were washed three times with PBS for 3 min, then perfused with 4% PFA. Fixed cells were permeabilized with 0.2% Triton X-100 in PBS for 10 min. After washing with PBS and blocking with 1% BSA for 1 h, the cells were incubated with antibodies against c-KIT at 4 µg/ml (Santa Cruz, sc-365504) or SM22α (Abcam, ab14106) at 5 µg/ml overnight under 4 °C. The corresponding fluorescent-labeled secondary antibody was added for 1h. Following re-staining with DAPI, the cells were observed with a laser confocal scanning microscope.

### *En-face* immunofluorescence staining

For *en-face* confocal laser scanning microscopy 18, the rats were perfused with 4% PFA in a HEPES buffer. Stented vessels were isolated and permeabilized with 0.2% Triton X-100 in PBS. Permeabilization and all of the following staining steps were performed in Eppendorf tubes. After rinsing with PBS-BSA (0.2% BSA in PBS) and immersion for 30 min in blocking solution (0.2% BSA and 5% goat serum in PBS or 12.5 mg purified goat IgG/mL), the tissue samples were incubated for 1 to 2 h in a PBS-BSA solution containing the primary antibody. In the single whole-mount staining experiments, primary monoclonal antibodies were used to detect the expression of c-KIT (Santa Cruz, sc-365504), or CD68 (Abcam, ab955). For single immunofluorescence labeling, donkey anti-mouse coupled to DTAF (dilution, 1/100, Jackson Laboratories) was used as the secondary antibody. After double staining with DAPI, the stented vessels were observed with a confocal laser scanning microscope.

### Immunohistochemical staining

Four-micron-thick plaque sample sections were incubated with a Total galactose-specific lectin 3 (MAC-2) antibody (Abcam, ab2785, 1:100), total chitinase 3-like 3 (YM-1) antibody (Abcam, ab192029, 1:500), AP-1 antibody (Abcam, ab40766, 1:100) or the proliferating cell nuclear antigen (PCNA) antibody (Abcam, ab29, 1:100) overnight at 4 **°C** after blocking with 1% goat serum. Slides underwent color development with DAB and hematoxylin counterstaining. Images were obtained with an optical microscope (Leica DMI6000B).

### Statistical analyses

Quantitative data were expressed as mean ± SEM. N indicates the number of independent experiments. Visual assessment was used to check for any lack of normality; because there was no evidence of this, one-way analysis of variance (ANOVA) followed by Tukey multiple comparison tests (for comparisons of >2 groups) or Student's t-test (for comparisons of 2 groups) were conducted. All statistical analyses were conducted using Prism version 4 (GraphPad Software, San Diego, California). For the qRT-PCR experiments, values are expressed as fold change. All statistical analyses used Graph Pad Prism v4 (GraphPad Software ^®^).

## Results

### M2 macrophages are adjacent to c-KIT^+^ VSMCs after intravascular stent implantation

316L bare metal stents (BMS) were implanted from the left iliac artery into the abdominal aorta of SD rats to identify the existence and localization of c-KIT^+^ VSMCs in the stent implantation sites. Immunofluorescence images showed that a large number of SM22α^+^ cells expressed c-KIT in the neointima at 7 days post-stenting. In comparison, at day 28 post-stenting, few c-KIT^-^+ SM22α^+^ cells were found surrounding the stent struts, while large numbers of c-KIT and SM22α double-positive cells were found around the stent wire in the neointima (**Figure S1A**). Strikingly, c-KIT and SM22α double-positive cells among the SM22α^+^ cells in the intima increased significantly from 13.64 ± 0.70% to 63.78 ± 4.12% at 7 and 28 days post-stenting, respectively (**Figure S1B**).

**Figure 1:**
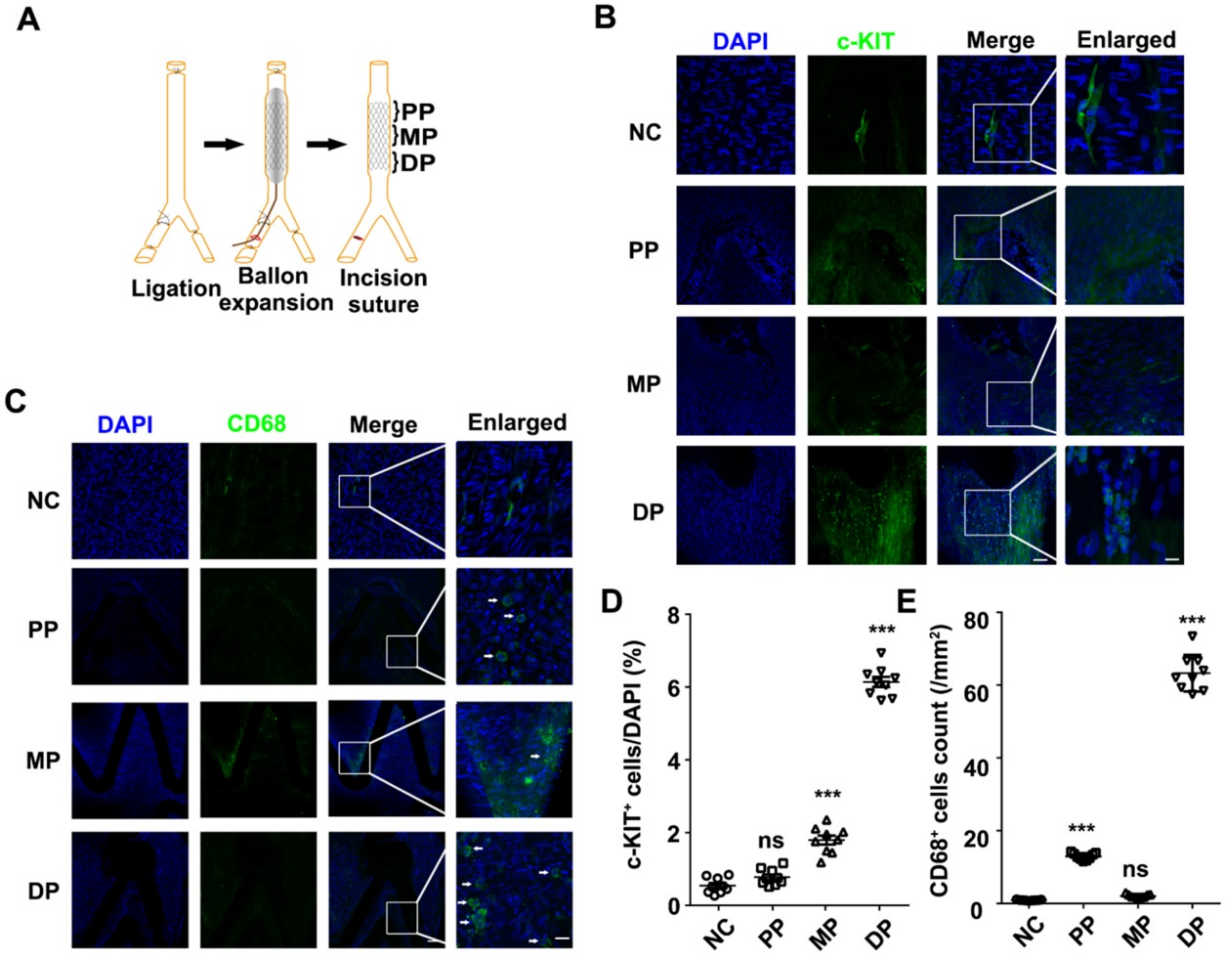
Both c-KIT^+^ and CD68^+^ cells were present in the distal part of the stented rat abdominal aorta vessels. (**A**) Schematic diagram of abdominal aortic stent implantation in rats; (**B**) 1 week after 316L BMS implantation, c-KIT^+^ cells were found at the DP of the stent segment compared to the NC, MP and PP. Areas under enhanced magnification correspond to the regions highlighted by the hatched rectangles. Scale bar: 100 µm (Merge); Scale bar:10 µm (Enlarged). (**C**) A week after implantation of the 316L BMS, the proportion of CD68^+^ cells in three positions of the stent segment were counted. The white arrow indicates the CD68^+^ cells. Scale bar: 100 µm (Merge) Scale bar: 10 µm (Enlarged). (**D**) Quantification of c-KIT^+^ cells in the three given fields of view per group. The results are representative of three independent experiments. (**E**) Quantification of CD68^+^ cells from three fields of view per group. N = 6 for each point in each group. Error bars are Mean ± SEM. *** *P <* 0.001 by one-way, ANOVA, ns means no difference. Full length of DP, MP, and PP. Abbreviations: NC, No-stented rat abdominal aorta vessels; DP, Distal Part of stented rat abdominal aorta vessels; MP, middle part of stented rat abdominal aorta vessels; PP, proximal part of stented rat abdominal aorta vessels.

To analyze the relationship between the repaired regions and c-KIT^+^ cells, we detected the c-KIT^+^ cells from the proximal to the distal parts of the stented vessels (**Figure 1A**). Through *en-face* staining, 6.14% of the c-KIT^+^ cells were found surrounding stent struts located in the distal part of the stented vessels (**Figures 1B and 1D**) compared with less than 1% and only 1.79% of c-KIT^+^ cells in the proximal and middle part of the stented vessels, respectively.

*En-face* staining of CD68 in the stented abdominal aortas was conducted to further study whether the macrophages were synchronized in appearance and localization with the c-KIT^+^ VSMCs. CD68^+^ cells were also found in the distal part of the stent implanted segments at 7 days post-stenting, while there were fewer CD68 positive cells in the normal abdominal aorta (**Figure 1C**). Almost 60 CD68 positive cells/mm^2^ were found in the distal part of the stent implanted vessels (**Figure 1E**). MAC-2 (marker for total macrophage) and YM-1 (marker for M2 macrophage) were detected in the distal part of the stented vessels by immunohistochemistry in order to further explore the macrophage type in the stented vessels. Both MAC-2^+^ cells and YM-1^+^ cells were found around the stent struts in the neointima. YM-1^+^ cells infiltrated deeper into the developing neointima and adjacent to the VSMCs at both 7 and 28 days post-stenting (**Figures S2A and S2B**). MAC-2^+^ cells were 8.77 ± 0.64% and 2.23 ± 0.20% in the developing neointima at 7 and 28 days post-stenting, respectively. The YM-1^+^ cells were 7.32 ± 0.45% and 1.43 ± 0.14% in the developing neointima at 7 and 28 days post-stenting, respectively (**Figure S2C**). These findings suggest that M2 macrophages may be a critical regulator in the c-KIT^+^ cells post-stent pathophysiological response to injury.

### Macrophages communicate with VSMCs through EVs

**Figure 2:**
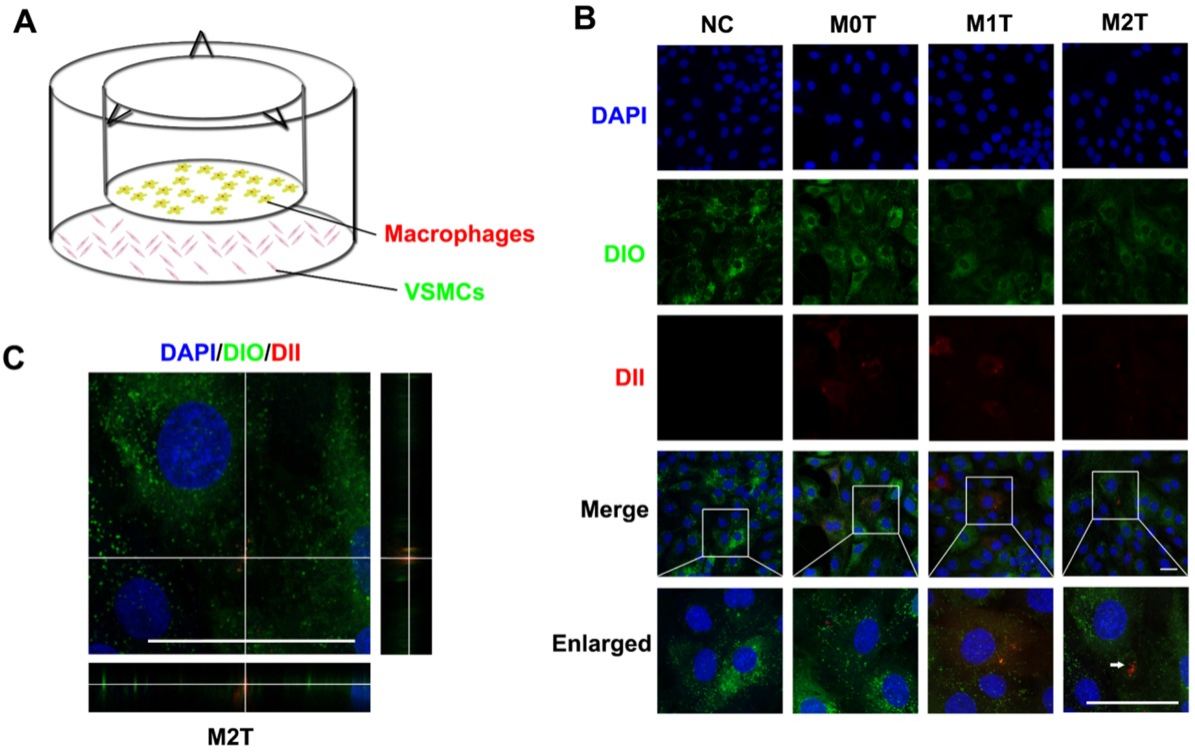
Microvesicles secreted by M2 macrophages can be incorporated into VSMCs. (**A**) Schematic diagram of co-culture of macrophages and VSMCs. (**B**) Naive (M0) macrophages, LPS+IFN-γ-stimulated (M1) macrophages and IL-4+IL-13-stimulated (M2) macrophages incubated with DII were co-cultured with A7R5 incubated with DIO in a Transwell (membrane pore = 0.4 µm) plate for 24 h. The white arrow indicates where DII accumulates in the VSMCs. Scale bar: 100 µm. (**C**) XZ and YZ side views of the A7R5 (green) co-cultured with M2 macrophage (red). Scale bar: 100 µm.

Next, we established a Transwell co-culture system to explore whether macrophages could enhance progenitor formation of VSMCs (**Figure 2A**). Naive macrophages (M0), LPS + IFN-γ stimulated macrophages (M1) and IL-4 + IL-13 stimulated macrophages (M2) stained with fluorescent dyes (red DII for the lipid membranes) were co-cultured with A7R5 stained with DIO in a Transwell (membrane pore = 0.4 µm) plate for 24 h. The morphology of the A7R5 cells was transformed from a long spindle to a polygon after co-culture with all kinds of macrophages (**Figure S4**).

The appearance of red fluorescent DII dyes in the A7R5 VSMCs demonstrated that the DII-stained membrane was delivered from the macrophages in the upper chamber of the Transwell to the recipient VSMCs seeded in the lower well. Compared with the M0 and M1 macrophage EVs, the VSMCs tended to uptake more of the EVs from the M2 macrophages (**Figures S5 and S6**). However, only RASMCs co-cultured with M2 macrophages showed an apparent aggregation of DII-stained cell membranes (**Figure 2B**). XZ and YZ views of the A7R5 confirmed that EVs from M2 macrophages were incorporated into the cells (**Figure 2C**).

### M2 macrophages upregulate the expression of c-KIT and decrease the cellular stiffness of VSMCs

**Figure 3:**
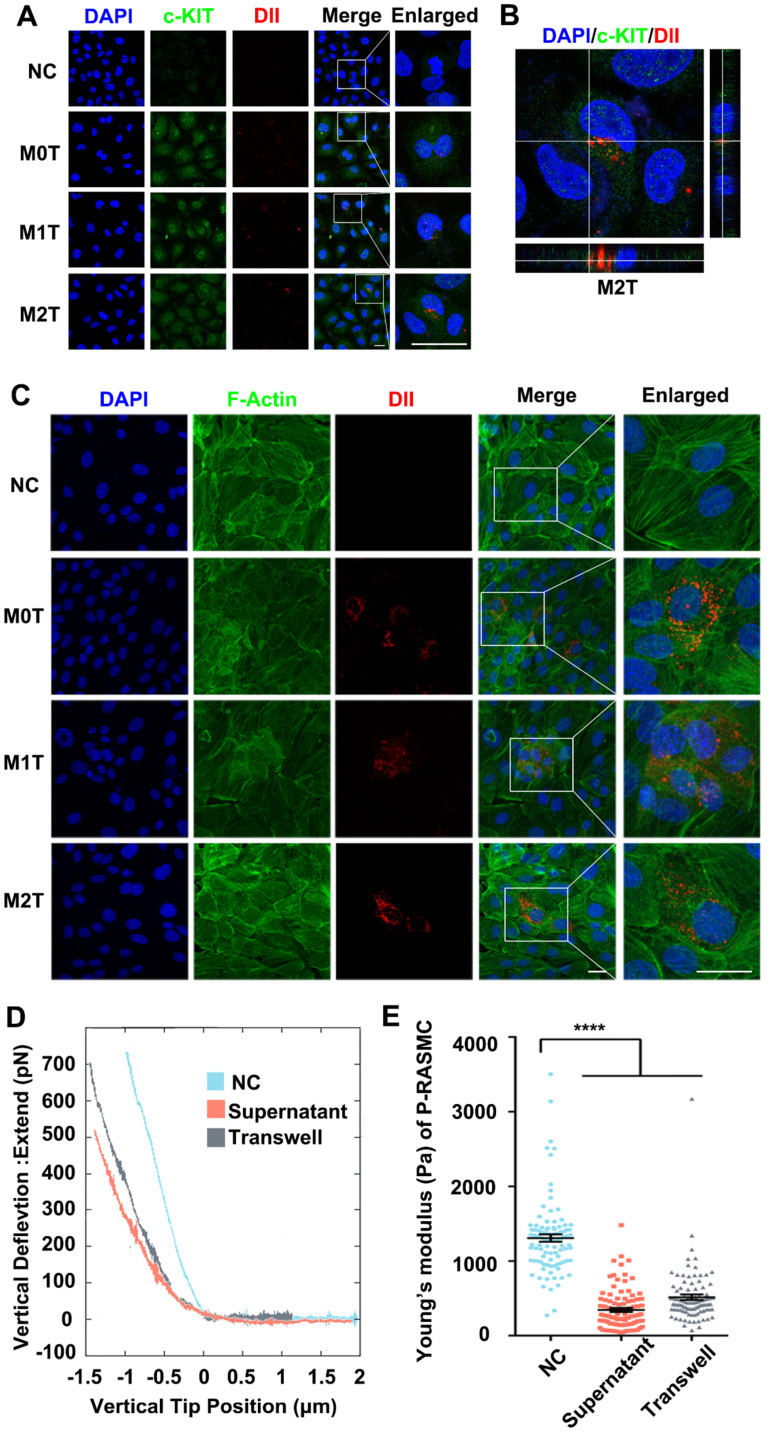
M2 macrophage EVs upregulate the expression of c-KIT of SMCs, broke up the F-actin skeleton of SMCs and decreased their stiffness. (**A**) Co-localization of c-KIT and macrophage microvesicle in RASMC, scale bar: 10 µm. (**B**) XZ and YZ sides view of the c-KIT (green) co-cultured with M2 macrophages (red), scale bar: 10 µm. (**C**) FITC-phalloidin dyeing of RASMC. 5 µg/ml FITC-phalloidin detects RASMCs' F-actin after 24 h of M0, M1 and M2 macrophage transwell co-culture stimulation, scale bar: 10 µm. (**D**) Representative force curves of force upon approach between the sphere and RASMC. Supernatant: RASMC treated with M2 macrophage supernatant; Transwell: RASMC co-cultured with M2 macrophages in a Transwell plate. (**E**) Quantification of resistance to mechanical deformation (stiffness) of the cells as in B for an applied load of 30 nN. At least ten cells were analyzed for each condition. Error bars represent Mean ± SEM. *P* values indicate the statistical significance of the differences of treatments versus the relevant control. **** *P <* 0.0001 by one-way, repeated-measures ANOVA (for 3 groups).

After detecting the stem cell marker c-KIT with immunostaining, the c-KIT of the inferior ventricular VSMCs was found to be upregulated in the co-cultured model (**Figure 3A**). Moreover, we analyzed the c-KIT, α-SMA and SM-22α protein levels after VSMC co-culturing with macrophages by western blotting. The data showed upregulated expression of c-KIT in RASMCs co-cultured with M2 macrophages but downregulated expression levels of α-SMA and SM-22α (**Figure S7**). Furthermore, compared with the M0 and M1 macrophage EVs treated group, FITC-phalloidin dyes of the RASMCs showed that the VSMCs that absorbed DII-stained M2 macrophages EVs had F-actin skeleton depolymerization (**Figure 3C**).

Stiffness changes can indirectly reflect the de-differentiation of VSMCs. We used colloidal probe force mode AFM to elucidate whether macrophages can regulate the stiffness of VSMCs. First, RASMCs were stimulated with macrophage conditioned medium (M2 macrophages supernatant: DMEM = 1:9) or by Transwell co-culture. Force curves of force upon approach between the sphere and an RASMC showed that the RASMC had a force curve with a lower slope under the M2 macrophage stimulation (**Figure 3D**). The JPK software analysis showed that the stiffness of the RASMCs was reduced significantly after the conditional medium stimulation or co-culture with M2 macrophages (**Figure 3E**).

Then, we pre-treated THP-1 macrophages with 10 µM GW4869, an inhibitor of exosome biogenesis/release, followed by IL-4 + IL-13 stimulation. We found that c-KIT expression of VSMCs co-cultured with M2 macrophages was not upregulated after GW4869 inhibited exosomes secretion by the macrophages (**Figure S8A**). At the mRNA level, the expression of *c-Kit*, *SM22α*, and *α-SMA* was restored to control levels after exosome inhibition (**Figure S8B**). These experiments showed that exosomes derived from M2 macrophages were responsible for the upregulation of c-KIT expression of the VSMCs after M2 macrophage stimulation.

### Exosomes secreted by M2 macrophages promote VSMCs softening and dedifferentiation

**Figure 4:**
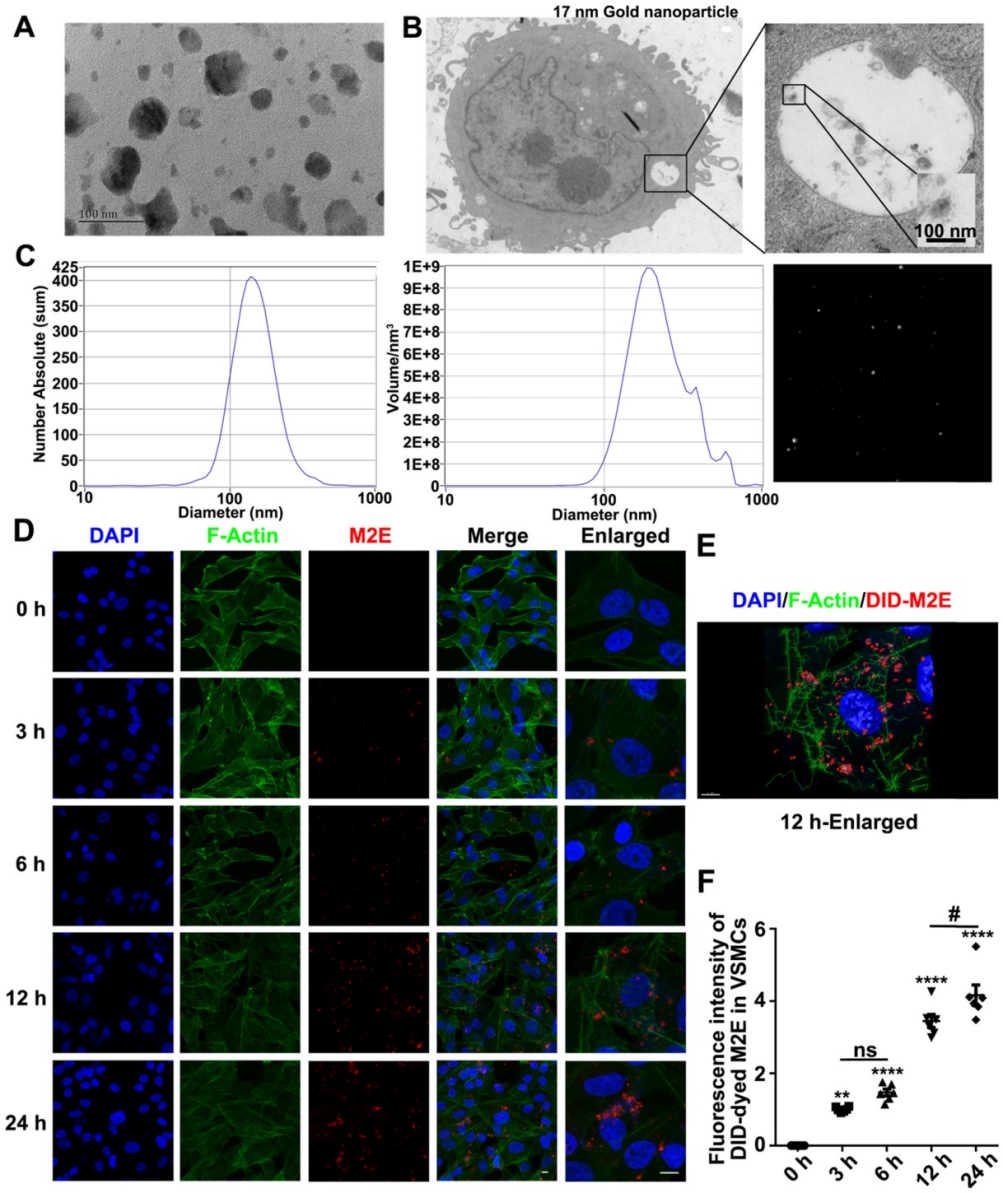
Exosomes mediate communication between M2 macrophages and VSMCs. (**A**) Isolated exosomes from M2 macrophages were examined by TEM. The scale bars indicate 100 nm. (**B**) TEM of p3-RASMCs treated with a 17 nm nanoparticle-labeled M2 macrophage exosome. The scale bars indicate 100 nm. (**C**) Size distributions of M2 macrophages-derived exosomes were identified using the NTA. (**D**) VSMCs were incubated with DID-labeled M2 macrophages-derived exosomes (100 µg/mL) for 3, 6, 12 and 24 h, and the exosome uptake by the VSMCs was detected (original magnification, ×630, scale bar: 25 µm; enlarged view, × 1890, scale bar: 10 µm). Arrows indicate the fusion of the membrane. (**E**) Confocal projection from 3D images of M2 macrophages exosome-internalized VSMCs after 12 h treatment with Imaris 7.2.3, scale bar: 25 µm. (**F**) Relative quantification of DID-dyed M2Es ingested into VSMCs, the fluorescence intensity of each group was normalized by the fluorescence intensity of M2 macrophages exosomes in VSMCs after 3 h (n = 6). **P < 0.01, **** *P <* 0.0001 versus the untreated group at the corresponding times and area, # *P <* 0.05 VAMCs treated with M2Es at 12 h versus the VAMCs treated with M2Es at 24 h; one-way, repeated-measures ANOVA.

TEM (**Figure 4A**) and NTA (**Figure 4C**) showed that the diameter of the exosomes extracted from the M2 macrophages supernatant was approximately 100 nm. To confirm that VSMCs directly engulf exosomes, exosomes labeled with 17 nm gold nanoparticles were incubated with the RASMCs for 12 h. The 17 nm gold nanoparticle labeled exosomes were engulfed by the VSMCs and could be detected by TEM (**Figure 4B**). *In vitro*, VSMCs absorbed more DID-dyed M2Es with the progression of time. At the same time, F-actin filaments of the VSMCs were depolymerized and rearranged (**Figure 4D**). 3D images of the M2Es-internalized VSMCs after 24 h treatment showed that depolymerization of F-actin took place around the DID-dyed M2Es (**Figure 4E**). The largest amount of DID-dyed M2Es was absorbed after 24 h based on the time serial statistical results (**Figure 4F**).

**Figure 5:**
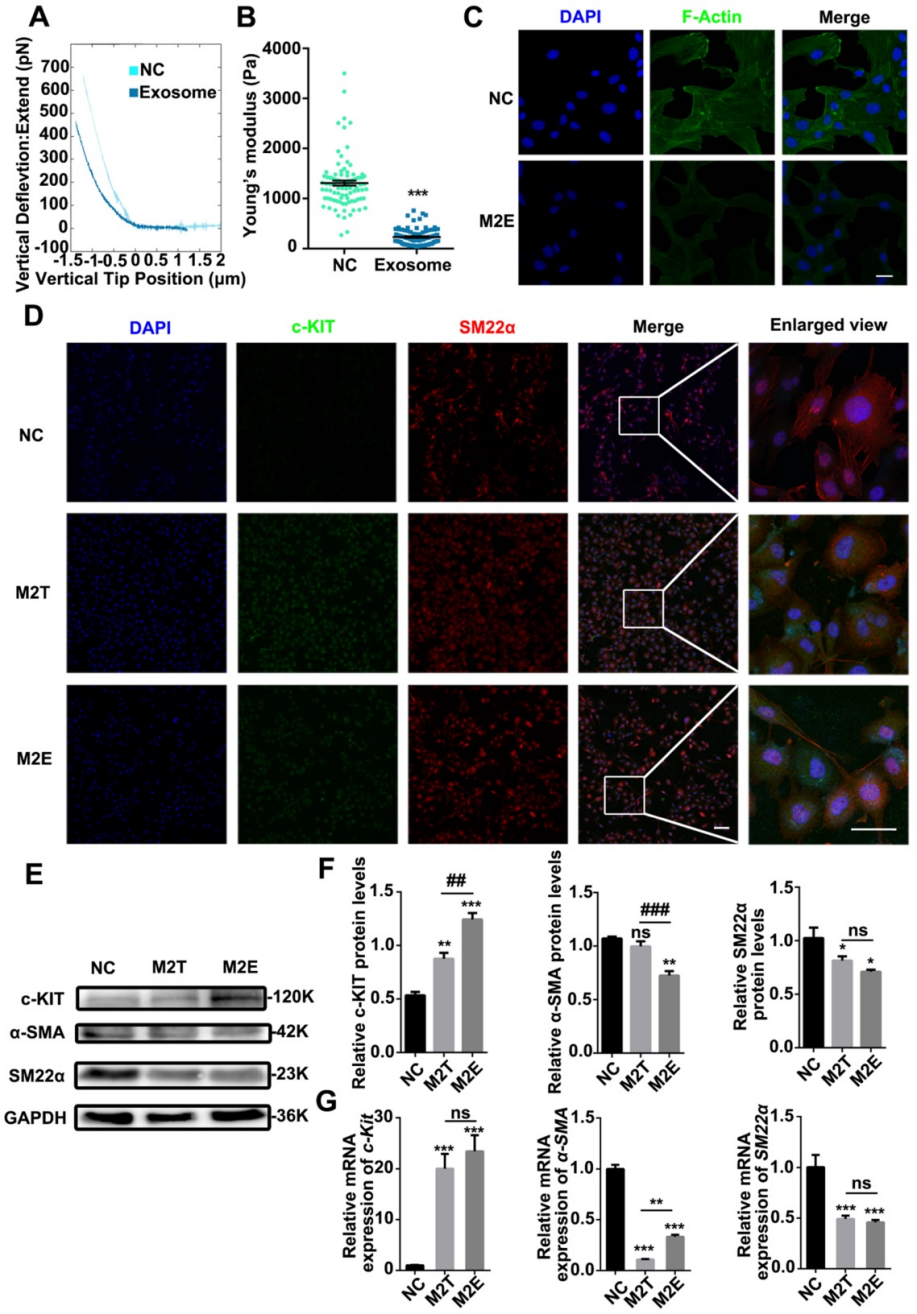
Exosomes secreted by M2 macrophages reduced the stiffness of SMCs and promoted SMC dedifferentiation. (**A**) Representative force curves of force upon approach between the sphere and PRASMCs (light green-blue) and PRASMCs treated with M2Es (dark green-blue). (**B**) Quantification of resistance to mechanical deformation (stiffness) of cells as in A at an applied load of 30 nN. Error bars represent Mean ± SEM. P values indicate the statistical significance of differences of treatments versus the relevant control. *** *P <*0.001 by unpaired, two-tailed Student's t-test. (**C**) FITC-phalloidin dyeing of PRASMC. Treatment with 5 µg/mL FITC-phalloidin detects PRASMCs' F-actin after 24 h of M2Es stimulation. Scale bar: 10 µm. M2Es: Primary RASMC treated with M2 macrophages exosome. (**D**) Quiescent RASMCs were grown in media free FBS (NC) and co-cultured with M2 macrophages in a Transwell (M2T) or exposed to exosomes derived from M2 macrophages (M2Es) for 24 h before the expression of c-KIT and SM22α were determined by immunofluorescence, scale bar: 10 µm. (**E**) Representative immunoblots for SMC differentiation markers (SM22α and α-SMA) and stem cell marker (c-KIT) in lysates from quiescent cells grown in FBS- free SMCM for 24 h. (**F**) The levels of c-KIT, SM22α and α-SMA were determined using specific antisera against these antigens. Images are representative of blots with similar results. Equal loading was confirmed by Ponceau S staining of the membranes and by measuring the constitutive GAPDH gene. The results are representative of three independent experiments. Error bars are Mean ± SEM. *P < 0.05, **P < 0.01, ***P < 0.001 versus the control group by one-way, repeated-measures ANOVA, ##*P <* 0.01, ###*P <* 0.001 versus the M2T-treated group at the corresponding times; one-way, repeated-measures ANOVA. (**G**) Quantification of relative mRNA expression of *SM22α*, *α-SMA* and *c-Kit* in the NC, M2T and M2Es. Error bars are Mean ± SEM. ***P < 0.001 vs. corresponding NC (one-way, repeated-measures ANOVA).

VSMCs were co-cultured with purified M2Es to clarify whether the M2Es induced progenitor strengthening and stiffness changes in the VSMCs. Compared with the control group, RASMCs co-cultured with M2Es were significantly softer than normal RASMCs. Young's modulus of untreated RASMCs and M2Es treated RASMCs were 1308 ± 49.94 Pa and 232.3 ± 14.76 Pa, respectively (**Figures 5A and 5B**). The VSMCs also developed a spherical morphology after exposure to M2Es. The ratio of long axis to short axis decreased from 5.620 ± 0.3676 to 2.161 ± 0.1652 (**Figure S9**). FITC-phalloidin staining showed that the F-actin skeleton of P3-RASMCs was broken after M2E treatment (**Figure 5C**).

Next, we hypothesized that M2Es could cause VSMC dedifferentiation at the protein level. After treatment with M2Es for 24 h, VSMC had a high level of c-KIT expression. Furthermore, the filamentous structure of SM22α in the VSMCs was no longer present under M2Es treatment (**Figure 5D**). We also examined the levels of c-KIT, SM22α and α-SMA proteins in untreated RASMCs and in M2Es stimulated RASMCs by western blotting. The results showed that c-KIT was highly upregulated in the M2Es stimulated group. SM22α and α-SMA were downregulated in the M2Es stimulated group (**Figure 5E and 5F**). The expression of *SM22α* and *α-SMA* were significantly reduced in the RASMCs after M2Es stimulation or co-culture with M2 macrophages, while the stem cell marker *c-Kit* was significantly increased. The other two markers *Sca-1* and *Oct-4* were not detected (**Figure 5G**). These data strongly indicate that M2Es are critical for the dedifferentiation of RASMCs.

### Exosomes secreted by M2 macrophages promote vascular therapy

**Figure 6:**
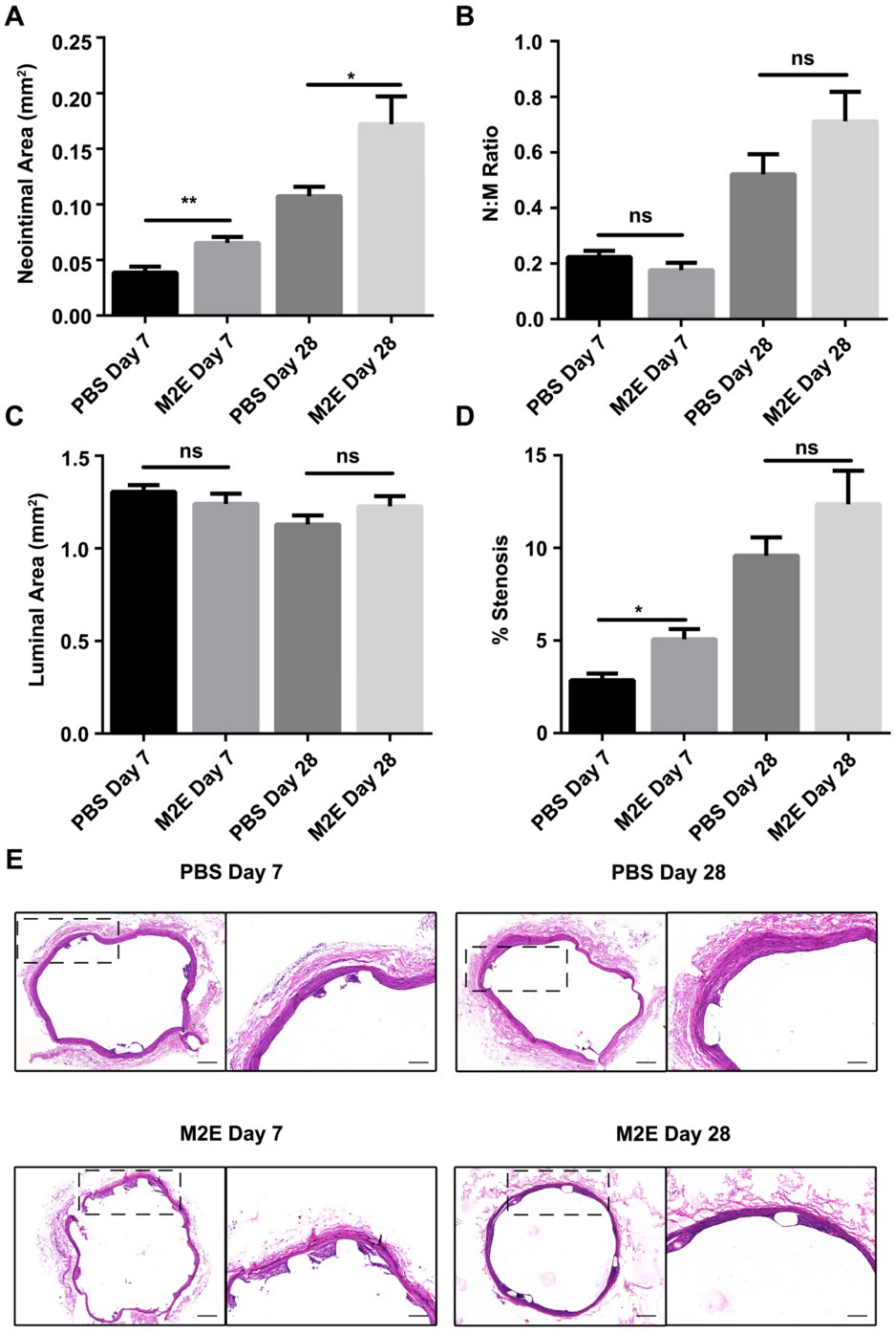
Morphometric analysis of stented abdominal aortas of rats with PBS treatment and M2Es treatment 7 and 28 days after stent placement. (**A**) neointimal area, (**B**) neointimal/media (N/M) ratio, (**C**) luminal area, and (**D**) percent stenosis. N = 7 for each group. *P <0.05, **P <0.01 versus PBS-treated groups (one-way, repeated-measures ANOVA). (**E**) Representative H&E stained sections from PBS-treated groups and M2Es-treated groups are seen 7 days and 28 days post-stenting. Scale bar: 200 µm.

**Figure 7:**
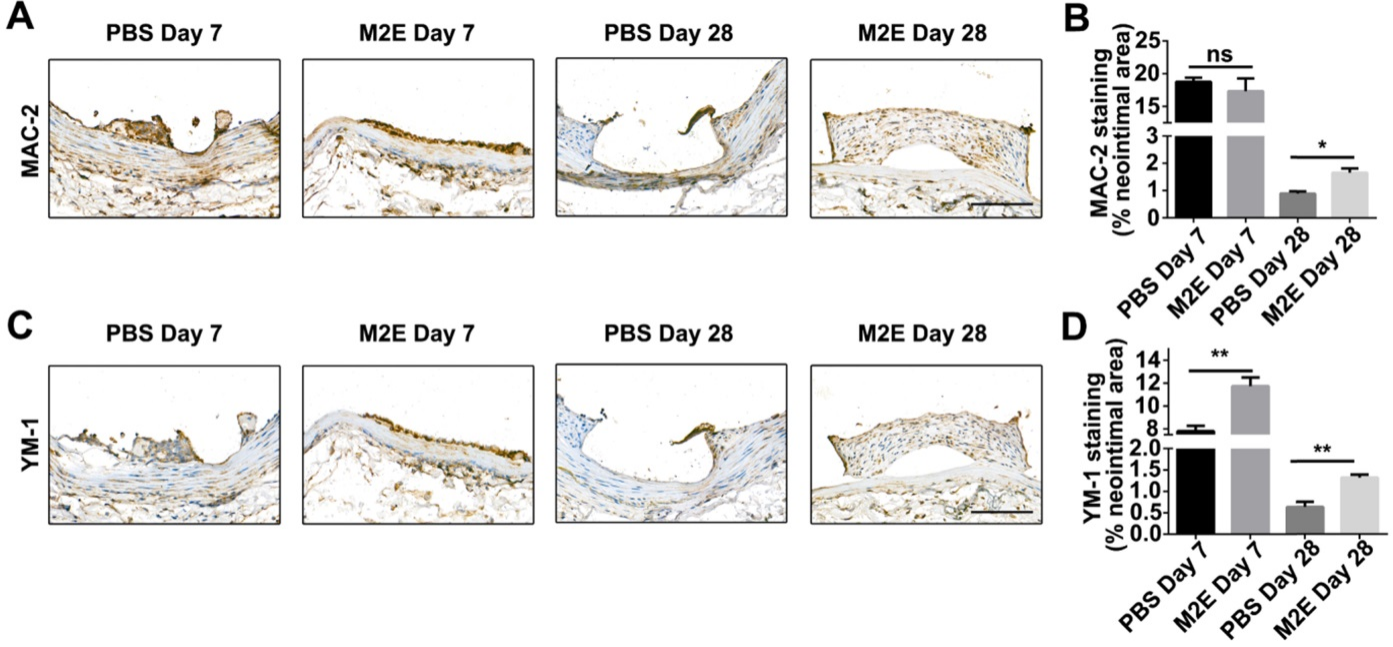
Inflammatory cells in neointimal lesions of M2Es-treated stented rats. (**A**) Representative images and (**B**) quantification of MAC-2 staining (% neointimal area) are seen in sections of stented vessels from PBS-treated and M2Es-treated rats at 7 and 28 days post-stenting (scale bar: 50 µm). (**C**) Representative images and (**D**) quantification of YM-1 staining (% neointimal area) are seen in sections of stented vessels from PBS-treated and M2Es-treated rats at 7 and 28 days post-stenting. Scale bar: 100 µm. *P <0.05; **P <0.01 versus PBS-treated rats (one-way, repeated-measures ANOVA).

We hypothesized that M2Es could enhance the vascular repair potency of VSMCs after abdominal aortic stent implantation in a rat model. Morphometric analysis found that M2Es-treated stent vessels had an increased neointimal area compared with PBS-treated controls at 7 days and 28 days (0.039 ± 0.006 vs. 0.065 ± 0.006, *P <* 0.01, day 7; 0.11 ± 0.009 vs. 0.17 ± 0.025, *P <* 0.05, day 28), but neointimal thickness, and the neointima-to-medial ratio did not differ significantly at 7 days and 28 days (**Figures 6A and 6B**, **Table S2**). A significant increment in percent stenosis in the M2Es-treated stent vessels compared with PBS-treated controls was also observed at 7 days (**Figures 6C and 6D**, **Table S2**). Furthermore, the total vessel area and medial area did not differ significantly at 7 days and 28 days (**Figure 6E**, **Table S2**). M2Es-treated stented rats contained greater numbers of MAC-2 macrophages in the neointima at 28 days compared with PBS-treated rats (0.89 ± 0.084% vs. 1.66 ± 0.16%; *P <* 0.05, day 28) (**Figures 7A and 7B, Table S2**), and had enhanced levels of YM-1^+^ macrophages at 7days and 28 days (7.81 ± 0.47% vs 11.74 ± 0.75%; *P <* 0.01, day 7; 0.63 ± 0.12% vs. 1.32 ± 0.072%; *P <* 0.01, day 28) (**Figures 7C and 7D, Table S2**). Thus, the M2Es resulted in an altered inflammatory cell phenotype within the stented vessels.

**Figure 8:**
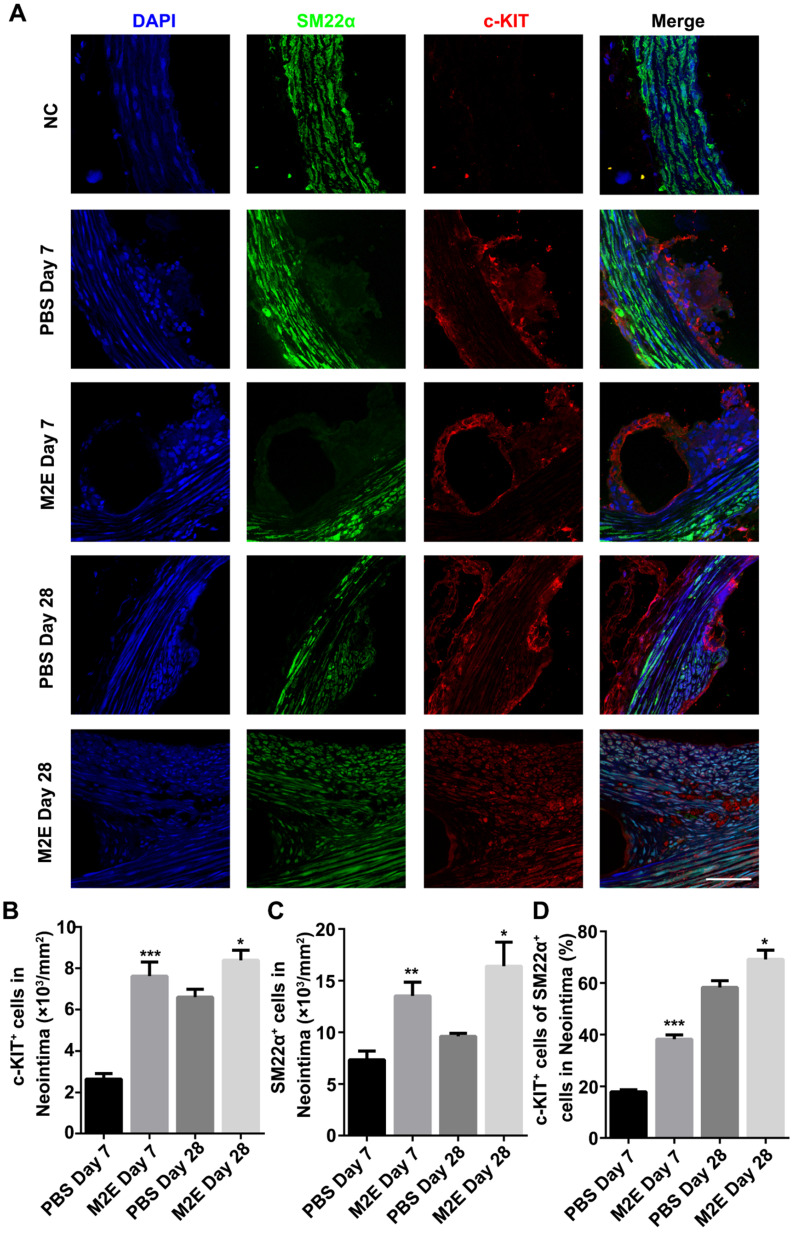
M2Es promote VSMC dedifferentiation in a rat abdominal aortic stent implantation model. (**A**) Immunofluorescence staining of paraffin sections at the site of the stented vessels. Scale bar: 25 µm. (**B**) The number of c-KIT^+^ cells was counted in the neointima. (**C**) The number of SM22α^+^ cells was counted in the neointima. (**D**) Percentage of c-KIT and SM22α double-positive cells was determined in the neointima at 7 and 28 days' post-stent placement. N = 7 for each group. *P <0.05, **P <0.01, ***P <0.001 versus the PBS-treated group at the corresponding times and for the same region (one way, repeated-measures ANOVA). Abbreviations: S, Stent; L, Lumen; M, Media; N, Neointima.

The neointimal lesions from the M2Es-treated rats contained significantly more c-KIT and SM22α double-positive cells at 7 and 28 days (**Figures 8A-C**, **Table S2**). Additionally, the M2Es-treated rats contained greater numbers of c-KIT^+^ with SM22α^low^ the SMCs in neointima compared with PBS-treated rats at 7 and 28 days (38.22 ± 1.67% vs 17.94 ± 0.81%, *P <* 0.001, day 7; 69.16 ± 3.51% vs 58.32 ± 2.56%, *P <* 0.05, day 28) (**Figure 8D**). No difference in cell dedifferentiation was observed after quantification of the cells in the media (**Figure S10, Table S2**). M2Es can accelerate the dedifferentiation of VSMCs after stent implantation. Immunohistochemistry showed that more PCNA^+^ cells were found in the neointima of the M2Es-treated group compared with that of the PBS-treated group (1.28 ± 0.22% vs 4.06 ± 0.79%, 7 days; 1.96 ± 0.34% vs 5.24 ± 0.55%, 28 days) (**Figures S11A and S11B, Table S2**).

### Increased AP-1 mediates the dedifferentiation of the RASMCs that absorbed M2Es

We compared the gene expression profile of RASMCs and RASMCs co-cultured with M2 macrophages by RNA-seq to further characterize RASMCs co-cultured with M2 macrophages. The top 10 functional pathways of significance (p-value) in the KEGG enrichment analysis results were visualized by line and bar graphs. Pathways relevant to this study were the MAPK signaling pathway, endocytosis, focal adhesion, cellular senescence, fluid shear stress and atherosclerosis and autophagy-animal (**Figure S12**).

**Figure 9:**
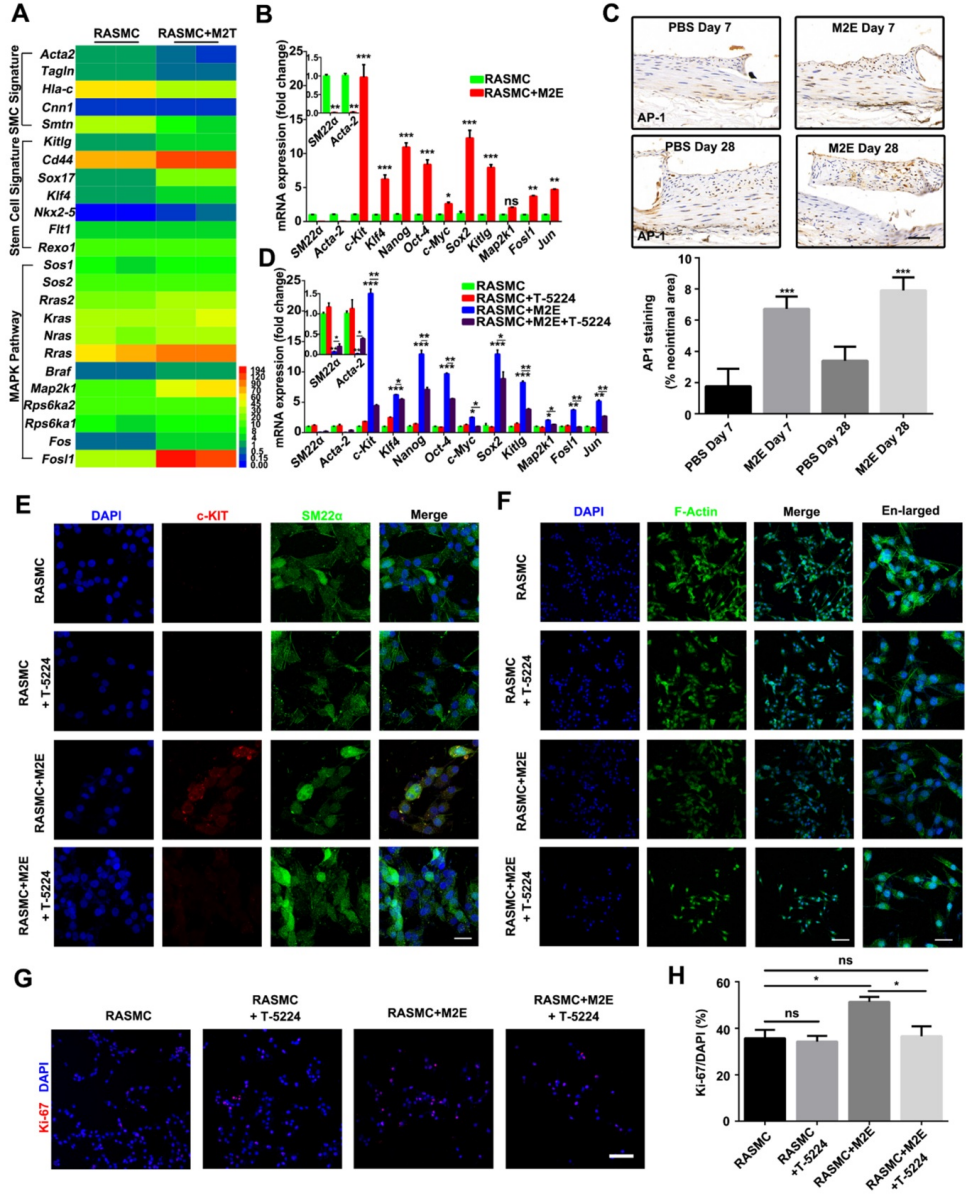
Activation of AP-1 in RASMCs facilitated VSMC dedifferentiation. (**A**) Heatmap displaying genes that were differentially expressed between RASMCs and RASMCs co-cultured with M2 macrophages. (**B**) qRT-PCR showing the expression of AP-1 target genes in RASMCs and M2Es-treated RASMCs. Data represent Mean ± SEM.*P < 0.05, **P < 0.01, ***P < 0.001 (Student's t-test). (**C**) Representative images and quantification of AP-1 staining (% neointimal area) are seen in sections of stented vessels from PBS-treated and M2Es-treated rats at 7 days and 28 days post-stenting (scale bar: 50 µm). Data represent Mean ± SEM. ***P < 0.001 (one way, repeated-measures ANOVA). (**D**) qRT-PCR verified the dedifferentiation and AP-1 pathways of M2Es-treated RASMC after AP-1 inhibition. Data represent Mean ± SEM.*P < 0.05, **P < 0.01, ***P < 0.001 (one-way, repeated-measures ANOVA). (**E**) Co-immunostaining for SM22α (green) and c-KIT (red) in RASMCs 24 h after culture in the presence of M2Es and/or T-5224 (10 µM). Nuclei were stained with DAPI (blue). Scale bar: 10 µm. *P < 0.05, **P < 0.01, ***P < 0.001 (one way, repeated-measures ANOVA). (**F**) FITC-phalloidin dyeing of RASMCs. Treatment with 5 µg/mL FITC-phalloidin detects RASMCs' F-actin 24 h after culture in the presence of M2Es and/or T-5224 (10 µM). Scale bar: 100 µm. Enlarged view scale bar: 10 µm. (**G**) Representative images of RASMCs labeled with Ki-67 (red) 24 h after different treatments. Scale bar = 100 µm. (**H**) Quantification of the percentages of Ki-67^+^ cells in RASMCs 24 h after different treatments. Values are Mean ± SEM (n = 6). *P < 0.05 by one-way, repeated-measures ANOVA.

Based on these findings, we speculated that the MAPK pathway plays an important role in the process of VSMC dedifferentiation. Genes upregulated in RASMCs co-cultured with M2 macrophages were associated with known stem cell signatures (*Kitlg*, *Cd44*, *Sox17*, *Klf4*, *Nkx-5*, *Flt1*, and *Rexo1*) (**Figure 9A**). On the other hand, genes associated with the SMC signature were downregulated in RASMCs co-cultured with M2 macrophages (**Figure 9A**). Although our RNA-seq did not detect that the* c-Kit* level changed in RASMCs co-cultured with M2 macrophages, qRT-PCR did find that *c-Kit* was also increased in M2Es-treated RASMCs (**Figure 9B**).

Importantly, bioinformatics analysis revealed that the expression of MAPK/AP-1 pathway genes (*Sos1*, *Sos2*, *Rras2*, *Kras*, *Nras*, *Rras*, *Braf*, *Map2k1*, *Rps6ka1*, *Rps6ka2*, *Fos*, and *Fosl1*) was significantly increased in RASMCs co-cultured with M2 macrophages, indicating AP-1 activity was increased in RASMCs co-cultured with M2 macrophages. Increased AP-1 activity in the RASMCs co-cultured with M2 macrophages might be responsible for the dedifferentiation of RASMCs induced by M2Es. qRT-PCR confirmed that *Fosl1* and other AP-1 dependent genes were highly expressed in the RASMCs that absorbed M2Es (**Figure 9B**). M2Es-treated rats highly expressed AP-1 in the neointima compared with PBS-treated rats at 7 and 28 days (1.76 ± 0.46% vs 6.72 ± 0.32%, *P <* 0.001, 7 days; 3.41 ± 0.37% vs 7.91 ± 0.34%, *P <* 0.001, 28 days) (**Figure 9C**).

Next, we used T-5224, a specific inhibitor of AP-1, to investigate whether AP-1 plays a functional role in the process of VSMC dedifferentiation. qRT-PCR confirmed that T-5224 potently inhibited AP-1 activity in M2Es-treated RASMCs (**Figure 9D**). MAPK/AP-1 pathway genes and stem cell signature related genes were downregulated, and SMC signature related genes were upregulated in RASMCs 24 h after culture in the presence of M2Es and T-5224 (10 µM) (**Figure 9D**). Immunofluorescence examination revealed that the expression of c-KIT in RASMCs stimulated by M2Es was significantly downregulated after AP-1 inhibition (**Figure 9E**). At the same time, the change of F-actin caused by M2Es also resumed after AP-1 was inhibited (**Figure 9F**). Moreover, T-5224 also significantly inhibited M2Es-induced RASMC proliferation (51.35 ± 2.09% vs 36.59 ± 4.28%, *P <* 0.05) (**Figures 9G and 9H**). In addition, the inhibition of AP-1 had no effect on the expression of caspase-3 in RASMCs (**Figure S14**).

## Discussion

The aim of this study was to investigate the roles of M2 macrophage-derived exosomes on VSMC dedifferentiation and stem cell acquisition, including the underlying mechanism after stent implantation. We made the unexpected observation that VSMCs strongly express c-KIT under the stimulation of exosomes secreted by M2 macrophages. Our findings also revealed that cell-to-cell communication between VSMCs and macrophages by M2 macrophage-derived exosomes (which increases the number of VSMCs with a c-KIT^+^ stem cell phenotype) may play a major role in early neointimal formation after stent implantation.

### M2 macrophages are involved in the neointima after stent implantation

Macrophage infiltration is quick and inevitable in the progression of neointimal formation. Previous *in vivo* studies suggested that macrophage infiltration is important in early thrombotic formation, VSMC migration, proliferation and apoptosis, and extracellular matrix production 19,20. Both M1 and M2 macrophages are found in the neointima after coronary stent implantation 21. It has been proposed that MCP-1-recruited macrophages induce intra aneurysmal tissue healing 22. Additionally, the scaffold could diminish the inflammatory reaction by reducing Tumor Necrosis Factor-α (TNF-α) secretion, upregulating the expression of M2 related genes, and increasing the number of M2 macrophages within the scaffold 23. Other findings support the hypothesis that additional M2 macrophages might cause less in-stent restenosis 21. Our discovery proves for the first time that CD23^+^ M2 macrophages infiltrated into the neointima and around the stent filaments. This result is consistent with a previous report that M2 macrophages mainly localize in the neointima in a gene knock-out mouse stent implantation model 21,24. Since M2 macrophages promote repair, it has been proposed that increasing M2 macrophage numbers in the wound may accelerate wound healing 25,26. Prostaglandin E2 hydrogel improves cutaneous wound healing via M2 macrophages polarization 27. Hence, M2 macrophages play an important role in vascular wound healing.

### Macrophage exosomes are essential for VSMC fate regulation

Exosomes secreted by M2 macrophages induce VSMC softening and dedifferentiation. An indirect co-culture system is suitable for simulating the cell-cell crosstalk, which could better simulate the early cellular behavior after stent implantation *in vivo*. Macrophage-derived exosomes can easily reach inflammatory sites and even cross the blood-brain barrier 28. Macrophage-derived foam cell released EVs were capable of transporting proteins to VSMCs, thus activating the ERK and Akt pathways in VSMCs and promoting VSMC migration and adhesion 29. Resistin-treated macrophage supernatant culture medium under different stimulations induces a shift from proliferation to apoptosis in VSMCs 30. Considering the pore size of Transwells and the diameter of the DII-stained membrane, we speculate that EVs secreted by macrophages may promote VSMC softening and dedifferentiation. In some special disease models, treatment of primary human pulmonary arterial smooth muscle cells with these EVs resulted in a significant increase in smooth muscle proliferation 31. However, they did not detect cell fate. *In vitro* Co-Cr alloy stent co-cultured systems, macrophages accelerate the proliferation of VSMCs by recruiting more macrophages 32. The phenotypes indicate the VSMCs underwent dedifferentiation with a change in function.

### VSMCs that engulfed exosomes participate in vascular therapy

VSMCs are a major neointimal constituent after stenting/vascular injury. In terms of the malignant proliferation of VSMCs caused by obesity, some studies showed that perivascular adipose tissue-derived extracellular vesicles enhanced VSMC proliferation and migration 33. At the same time, mesenchymal stem cells (MSCs) derived exosomes suppressed VSMC proliferation and migration and established neointimal formation in rat carotid arteries following angioplasty 29. In this paper, we showed that VSMCs rapidly cover the area of intimal injury and stent filaments and block contact with the blood at day 7 after stenting. A small subpopulation of resident VSMCs with a stem cell phenotype may be stimulated to proliferate and accumulate into the tunica intima in response to chronic injury or atherosclerotic pathogeny 34-36.

Rare c-KIT^+^ cells are observed in the sub-endothelial and outer portion of adult rat aortic media. Our results show that c-KIT expression was upregulated in VSMCs after stent implantation. Studies in rodent models indicated that the expression of c-KIT might protect VSMCs against apoptosis and maintain intimal hyperplasia after vascular injury 37. Previous studies have shown that stem cell differentiation or iPS formation was accompanied by changes in cell stiffness 10,19,38. In this study, we found that the VSMCs' stiffness was significantly decreased under the stimulation of macrophages. Therefore, stem cell factor (SCF)/c-KIT signaling may play a role in VSMC pathophysiology during the development of intimal hyperplasia 39.

The novel and most interesting finding of the present study is that co-culture of VSMCs with M2Es resulted in the dedifferentiation of VSMCs. This suggests that M2Es induce an increase of VSMCs with a c-KIT^+^ stem cell phenotype to induce repair in injured blood vessels. Exogenous M2Es can increase the stemness of VSMCs after vascular injury in a short time and strengthen their vascular healing potency. Exosome-based therapy in cancer and organ abnormality and mesenchymal stem cell transplantation have received increasing attention in recent years 40,41. We provide evidence that exogenous M2Es induce vascular repair through VSMC expressing c-KIT. It is possible that c-KIT suppresses nuclear factor kappa-B (NF-κB)-regulated pathways in VSMCs to prevent their pro-inflammatory transformation 9,12.

AP-1, a transcription factor, participates in the regulation of proliferation, migration and cell cycle progression in VSMCs 42-44. Additionally, the activation of AP-1 plays an important role in the process of aging 45, atherosclerosis 46,47, and vascular remodeling 48-50. According to the latest research, AP-1 plays an essential role in the cardiomyocyte response to injury by regulating chromatin accessibility changes, thereby allowing the activation of gene expression programs that promote cardiomyocyte dedifferentiation, proliferation, and protrusion into the injured area 51. In our study, the dedifferentiation of VSMCs after stent implantation was accompanied by the activation of AP-1. *In vitro*, when AP-1 was inhibited, the dedifferentiation of VSMCs induced by M2Es was also inhibited.

In conclusion, this study revealed cell-cell communication between M2 macrophages and VSMCs that promotes re-endothelialization after stent implantation. Thus, our study reveals a new mechanism of M2E-induced re-endothelialization: M2Es increase VSMC dedifferentiation via an M2 macrophages → exosomes → MAPK (AP-1) pathway. The challenge for future studies will be to identify the component in the M2 macrophage exosomes that induces phenotypic transitions of VSMCs.

## Supplementary Material

Supplementary figures and tables.

## Abbreviations

VSMCs: vascular smooth muscle cells
A7R5: rat aortic smooth muscle cells
RASMC: rat aorta smooth muscle cells
THP-1: human leukemic monocyte
ETO: ethylene oxide
EVs: extracellular vesicle
PBS: phosphate buffer saline
M2E: exosomes derived from M2 macrophage
H&E: hematoxylin and eosin
RPMI: roswell park memorial institute
PMA: phorbol 12-myristate 13-acetate
BSA: bovine serum albumin
LPS: lipopolysaccharide
IFN-γ: Interferon-gamma
IL-4: interleukin-4
IL-13: interleukin-13
DMEM: dulbecco's modified eagle's medium
FBS: fetal bovine serum
NTA: nanoparticle tracking analysis
PAGE: polyacrylamide gel electrophoresis
PVDF: polyvinylidene fluoride
TBST: tris-buffered saline Tween-20
HRP: horseradish peroxidase
ECL: electrochemiluminescence
AFM: atomic force microscopy
TEM: transmission electron microscopy
FITC: fluorescein isothiocyanate
DII: 1,1′-dioctadecyl-3,3,3′,3′-tetramethylindocarbocyanine perchlorate
DIO: 3,3′-dioctadecyloxacarbocyanine perchlorate
DID: 1,1-dioctadecyl-3,3,3,3-tetramethylindodicarbocyanine
SMCM: smooth muscle cell medium
TNF-α: tumor necrosis factor-α
MCP-1: monocyte chemotactic protein-1
iPS: induced pluripotent stem cells
SCF: stem cell factor
NF-κB: nuclear factor kappa-B
PCNA: proliferating cell nuclear antigen
MAC-2: total galactose-specific lectin 3
YM-1: total chitinase 3-like 3
AP-1: activator protein 1
