# Supplementary material for: M2 macrophage-derived exosomes promote the c-KIT phenotype of vascular smooth muscle cells during vascular tissue repair after intravascular stent implantation
Source: Theranostics. 2020 Aug 29;10(23):10712–28. doi: 10.7150/thno.46143 (PMC7482821; doi:10.7150/thno.46143)
Supplement: Supplementary file 1 — Supplementary figures and tables. [file thnov10p10712s1.pdf]

Supplementary Materials

M2 macrophage-derived exosomes promote the c-KIT phenotype of vascular smooth muscle cells during vascular tissue repair after intravascular stent implantation

Wenhua Yan[1], Tianhan Li[1], Tieying Yin[1], Zhengjun Hou[1], Kai Qu[1], Nan Wang[3], Colm Durkan[3], Lingqing Dong[4], Juhui Qiu[1, *], Hans Gregersen[1,2, *], Guixue Wang[1, *]

[1] Key Laboratory of Bio-Rheological Science and Technology, State and Local Joint Engineering Laboratory for Vascular Implants, Bioengineering College of Chongqing University, Chongqing, China

[2] GIOME, Department of Surgery, the Chinese University of Hong Kong, Hong Kong, China

[3] The Nanoscience Centre, University of Cambridge, Cambridge, UK

[4] Cavendish Laboratory, University of Cambridge, Cambridge, UK

* Correspondence to: Dr. Juhui Qiu, Email: jhqiu@cqu.edu.cn; Dr. Hans Gregersen, Email: hag@giome.org; Prof. Guixue Wang, Email: wanggx@cqu.edu.cn

Stent implantation in rat aortas

Forty-six 12-14 weeks old Sprague-Dawley (SD) male rats weighing 400–500 g were purchased from the Third Military Medical University in Chongqing, China. The animals were divided into two groups, including 10 rats in the sham-operated group and 36 rats in the 316L bare-metal stents (BMS) abdominal aorta implantation group. The animal experiments were in compliance with the Animal Ethics Committee of Chongqing University. Every process followed the ethical guidelines for experimental animals. All animals were fed aspirin + clopidogrel -incorporated food (5 mg/kg/day, Bio-Serv, Frenchtown, NJ) three days before surgery to the end of the study period in order to prevent thrombosis after stent implantation. All bare metal stents were pre-mounted on VasoTech® Miniature balloon catheters (1.5 mm × 15 mm, VasoTech, Inc.) and sterilized with Ethylene Oxide (ETO) for 60 min before implantation.

Briefly, all animals fasted for 12 h prior to surgery. After the animal was fully anesthetized with constant inhalation of a mixture of oxygen/isoflurane (1.5:2 pressure/pressure), the left iliac artery was exposed. The stent was inserted into the abdominal aorta 10 mm above the bifurcation through a left iliac arterial incision and deployed by inflating the balloon catheter to 10 ATM for 30 s. The balloon catheter was deflated to maintain negative pressure for 30 s. The process was repeated three times to fully deploy the stent. The deflated catheter was then with-drawn slowly while leaving the stent in place. The artery was sutured with 9–0 bio-absorbable sutures, and gentamicin (50 mg/kg) was administered to all animals for three days following the surgery.

To deliver M2E into the stented abdominal aortic and to avoid any potential systemic side effects, we applied an established local delivery model via pluronic gel F-127, as described in previous reports with little modification. 28 stented rats (PBS-7d, PBS-28d, M2E-7d and M2E-28d, n = 7 for 4 groups, respectively) and 7 normal rats. Briefly, immediately after stent implantation, 50 μg M2E or PBS preloaded into 50 μL 20% pluronic gel F-127 (Sigma) at 4 °C was applied locally to the adventitia around stented artery segments. Then, M2E (10 μg) in PBS or vehicle (PBS) were injected tail intravenously every three days until the rats were sacrificed.

Animals were euthanized after 7 or 28 days by intravenous overdose of euthatal, 100 U/mL heparin sodium was pressurized to remove excess blood cells and 4% paraformaldehyde (PFA) was added to maintain vascular morphology. Stented vessels carefully removed, and vessels were fixed in 4% PFA for histological analysis.

Cell Culture

THP-1 human acute monocytic leukemia cells were kindly provided by the Stem Cell Bank, Chinese Academy of Sciences. Cells were cultured in RPMI-1640 containing 10% FBS, glucose (11 mM), L-glutamine (4 mM), penicillin (100 U/mL), and streptomycin (100 μg/mL) and incubated at 37 °C in a humidified atmosphere of 5% CO_2 and 95% air. For all experiments, THP-1 cultured by passage were diluted into 1×10^6/mL,

inoculated in a 35 mm culture dish, and cultured in serum-free RPMI-1640 medium containing phorbol-12-myristate-13-acetate (PMA) (Sigma-Aldrich, P8139, 100 ng/ml) and 0.3% bovine serum albumin (BSA) for 72 h to induce differentiation. Morphological observation was carried out under light microscopy to identify whether the cells differentiated into macrophages. To mimic a pro-inflammatory environment and consequently promote M1 or M2 polarization, M1 cells were stimulated with lipopolysaccharide (LPS) (Sigma, #8630, 1 μg/mL) and interferon-γ (IFN-γ) (Novus Biologicals, #NBP2-34992, 20 ng/mL). For M2 polarization, cells were stimulated with IL-4 (Novus Biologicals, #NBP2-34896, 10 ng/mL) and IL-13 (Novus Biologicals, #NBP2-35018, 10 ng/mL) was added back with DMSO (carrier) for 48 h.

The phagocytic preference of VSMCs for EVs of pro-inflammatory and anti-inflammatory macrophages

We labeled M0 macrophages with DID, M1 macrophages with DIO and M2 macrophages with DII, and washed three times with PBS for 5 min each time. Then, after 12 h of serum-free culture, we cultured them in exosome-free 1640 medium for 48 h to obtain supernatant containing exosomes. Cell debris was removed by low-speed centrifugation and larger particles were removed by 0.22 μm sterile filter. We added the supernatants of macrophage culture medium of equal volume of M0, M1 and M2 into VSMCs (10% of the total medium). After 10 min of nucleation with DAPI, we observed the preference ingestion of VSMCs for EVs of different types of macrophages under confocal microscopy (Supporting Information Figure S5 and S6).

Supplementary tables

Table S1. Primers used in real-time qPCR reactions

		Sequence, 5'-3'	
Gene	Protein	Forward	Reverse
Gapdh	GAPDH	CGTGTTCCTACCCCCAATGT	TGTCATCATACTTGGCAGGTTTCT
Sm22α	SM22α	CGCGAAGTGCAGTCCAAAAT	TTGGAGCCATCAGGGTACAG
Acta2	ACTA2	CAATGAGCTTCGTGTTGCCC	CATAGAGAGACAGCACCGCC
c-Kit	c-KIT	TGAATGGCATGCTCCAATGTGTGG	ACATCCACTGGCAGTACAGAAGCA
Oct-4	OCT-4	ATGCATTCAAACTGAGGTGCCTGC	AACTTCACCTTCCCTCCAACCAGT
Sca-1	SCA-1	TGTTTGAAATGTGCGGAGTGT	ACACAGCGGAAACACTCGAT
Klf4	KLF4	TTCACCTATCCGATCCGGGC	TGTACACCGGGTCCAATTCTG
Nanog	NANOG	GAGATGCCTCACACGGAGAC	AGCTGGGTGGAAGAGAACAC
C-myc	C-MYC	CCTTCGGGGAGACAACGAC	CGAGAAGCCGCTCCACATAC
Sox2	SOX2	TTTGTCGGAGACGGAGAAGC	GGGCAGCGTGTACTTATCCT

Kitlg	KITLG	TGAAGGGATCTGCAGGAATCG	CCGGGGACATATTTGAGGGT
Map2k1	MAP2K1	TTCAAGGTCTCCCACAAGCC	TCTCGCCATCGCTGTAGAAC
Fosl1	FOSL1	CGTTGTGAAGACCATGACAGG	TGTATCAGTCAGCTCCCTCCT
Opn	OPN	TCCAACGAAAGCCATGACCA	GCAGGTCCGTGGGAAAATCA
Elastin	ELASTIN	CTTAAGCCAGTTCCCGGAGG	TGCAGACACTCCTAAGCCAC
Mgp	MGP	TTTGTGTTATGAATCACATGAAAGC	GTGGACAGGCTTAGAGCGTT
Jun	JUN	GAGCTGGAGCGCCTGATAAT	CCCTCCTGCTCATCTGTCAC

Table S2. Morphometric analysis of stented rat vessels.

	PBS-1W	M2E-1W	p-Value	PBS-1M	M2E-1M	p-Value
MAC-2 staining (% neointimal area)	18.77 ± 0.64	17.33 ± 1.93	ns	0.89 ± 0.084	1.66 ± 0.16	p<0.05
YM-1 staining (% neointimal area)	7.81 ± 0.47	11.74 ± 0.75	p<0.01	0.63 ± 0.12	1.32 ± 0.072	p<0.01
PCNA staining (% neointimal area)	1.28 ± 0.22	4.06 ± 0.79	p<0.01	1.96 ± 0.34	5.24 ± 0.55	p<0.01
Total vessel Area, mm^2	1.57 ± 0.051	1.60 ± 0.062	ns	1.55 ± 0.059	1.64 ± 0.066	ns

Neointimal (N) Thickness	-	-	-	0.058±0.007	0.053±0.014	ns
Neointimal Area, mm^2	0.039 ± 0.006	0.065 ± 0.006	p<0.01	0.11 ± 0.009	0.17 ± 0.025	p<0.05
Medial (M) Area, mm^2	0.22 ± 0.018	0.25 ± 0.019	ns	0.22 ± 0.020	0.24 ± 0.017	ns
N/M ratio	0.18 ± 0.028	0.22 ± 0.022	ns	0.53 ± 0.073	0.72 ± 0.11	ns
Lumen Area, mm^2	1.31 ± 0.037	1.24 ± 0.056	ns	1.13 ± 0.049	1.23 ± 0.055	ns
% Stenosis	2.85 ± 0.37	5.07 ± 0.55	p =0.01	9.58 ± 0.99	12.36 ± 1.81	ns
$SM22a^+$ cells /Neointimal Area($\times 10^3/mm^2$)	7.36 ± 0.84	13.52 ± 1.34	p<0.01	9.6 ± 0.30	16.40 ± 2.32	p<0.05
$c\text{-}KIT^+$ cells / Neointimal Area ($\times 10^3/mm^2$)	2.65 ± 0.26	7.62 ± 0.68	p<0.001	6.61 ± 0.38	8.38 ± 0.49	p<0.05
c-KIT+SM22a double-positive/$SM22a^+$ in Neointima (%)	17.94 ± 0.81	38.22 ± 1.67	p<0.001	58.32 ± 2.56	69.16 ± 3.51	p<0.05
$SM22a^+$ cells/ Medial Area($\times 10^3/mm^2$)	7.731 ± 1.08	3.69 ± 0.63	p<0.05	5.53 ± 0.26	6.39 ± 0.69	ns
$c\text{-}KIT^+$ cells / Medial Area ($\times 10^3/mm^2$)	0.29 ± 0.13	0.64 ± 0.21	ns	0.30 ± 0.13	0.34 ± 0.046	ns
c-KIT+SM22a double-positive/$SM22a^+$ in Media (%)	3.67 ± 0.73	2.92 ± 0.71	ns	2.5 ± 0.89	5.55 ± 0.84	p<0.05

-: The neointima was too thin, neointimal (N) thickness can't be counted at 7 days.

Supplementary figures

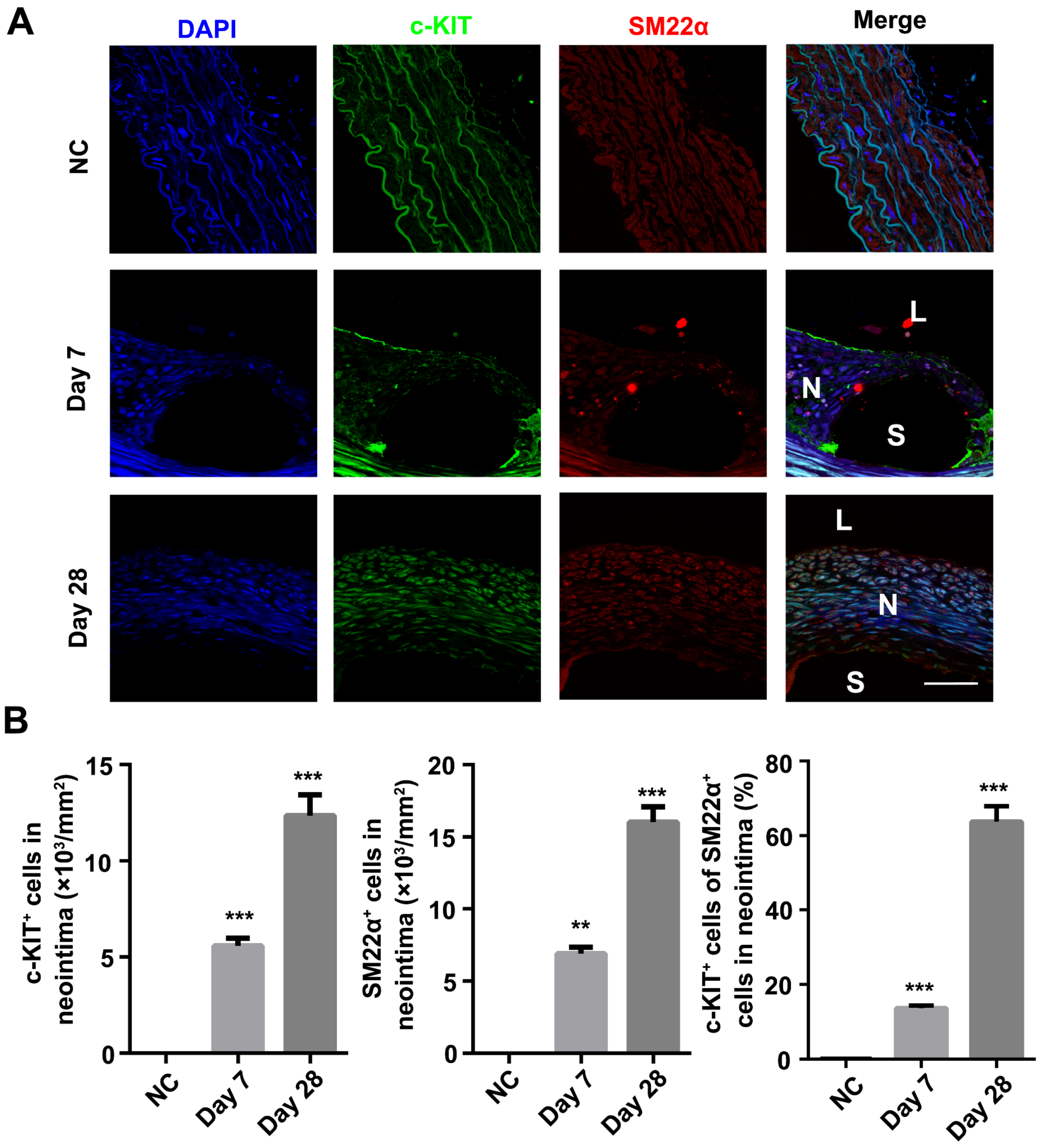

Figure S1. (A) Immunofluorescence for c-KIT and SM22α in normal rat abdominal aorta vessels and in BMS-stented vessels at 7 and 28 days post-stenting (representative images, n = 6). Scale bar = 25 μm. (B) The numbers of c-KIT^{+} and SM22α^{+} cells were counted in neointima, and the proportion of c-KIT^{+} cells to SM22α^{+} cells in neointima. **$p < 0.01$ ***$p < 0.001$ versus normal rat abdominal aorta vessels in each group (one-way, repeated-measures ANOVA). S = Stent; L = Lumen; N = Neointima.

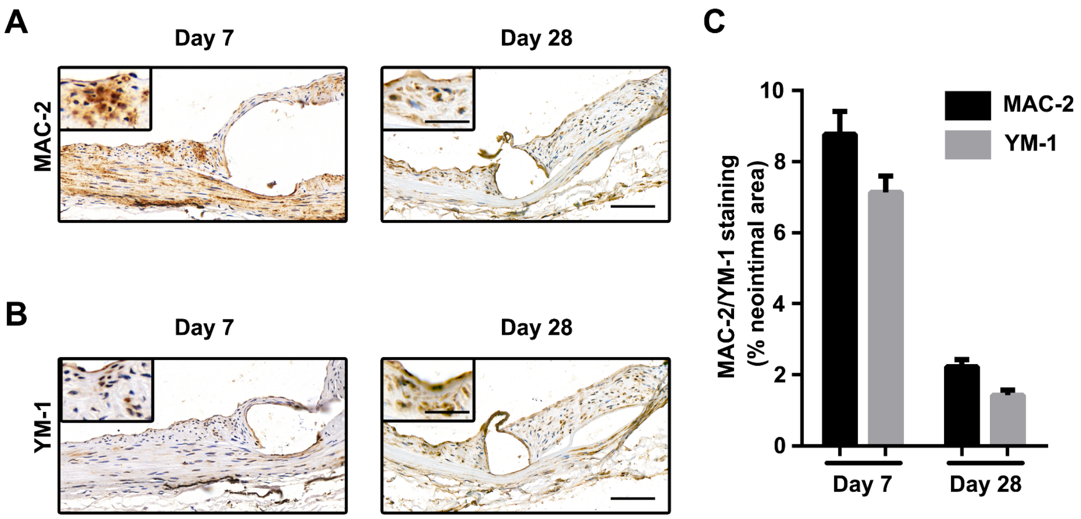

Figure S2. YM-1^{+} cells infiltrated into the neointima after 316L stent implantation. (A and B) Immunohistochemistry for MAC-2 and YM-1 in the BMS-stented vessels at 7 and 28 days post-stenting (representative images, n = 6). Scale bar =100 μm, scale bar = 25 μm (En-larged view). The enlarged area represents MAC-2^{+} and YM-1^{+} cells. (C) Quantification of MAC-2 and YM-1 (% neointimal area) were seen in sections of stented vessel at 7 and 28 days post-stenting.

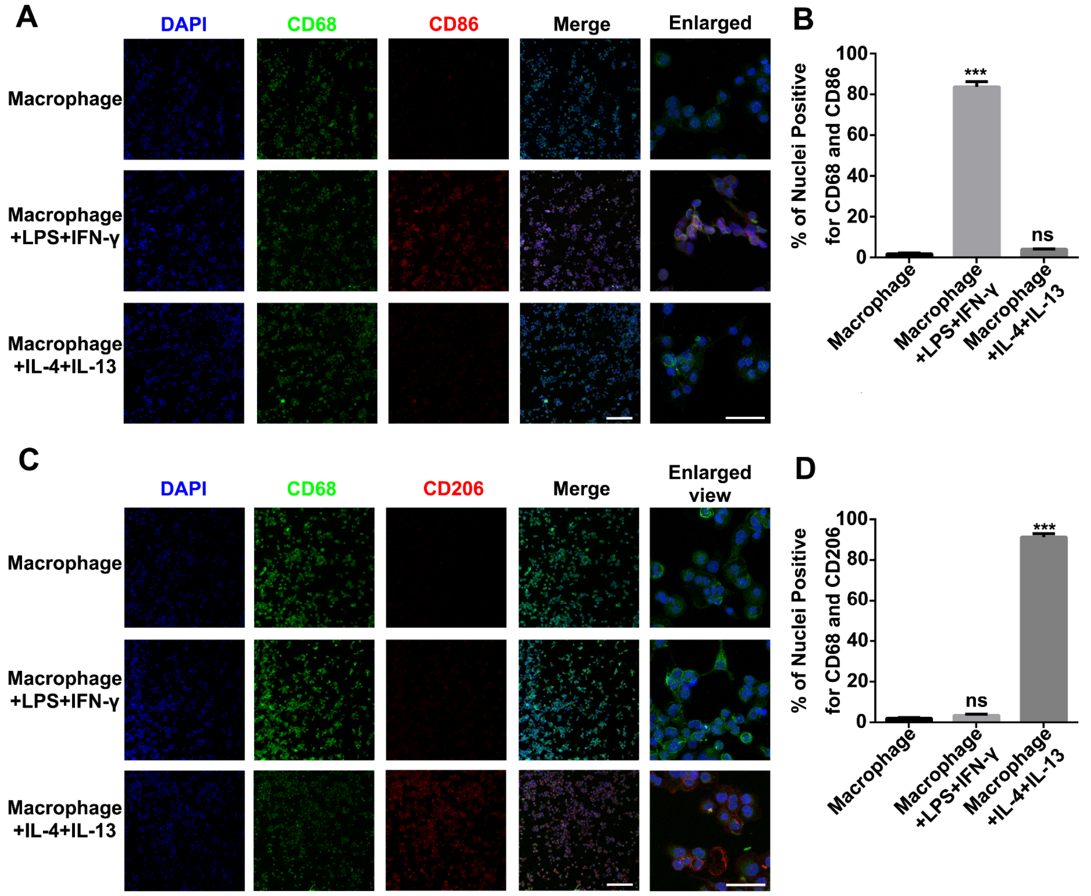

Figure S3. Identification of pro-inflammatory and anti-inflammatory macrophages after different stimuli. (A and C) Immunofluorescence for CD68+CD86 and CD68+CD206 of macrophages after LPS (1 μg/mL) +IFN-γ (20 ng/mL) and IL-4 (10 ng/mL) +IL-13 (10 ng/mL) stimulated (representative images, n = 3). Scale bar = 100 μm (100 ×), scale bar = 10 μm (630 ×). (B and D) The numbers of CD68+CD86 double-positive and CD68+CD206 double-positive cells were serially counted. ***P < 0.001 versus unstimulated macrophages at the corresponding area (one way, repeated-measure ANOVA).

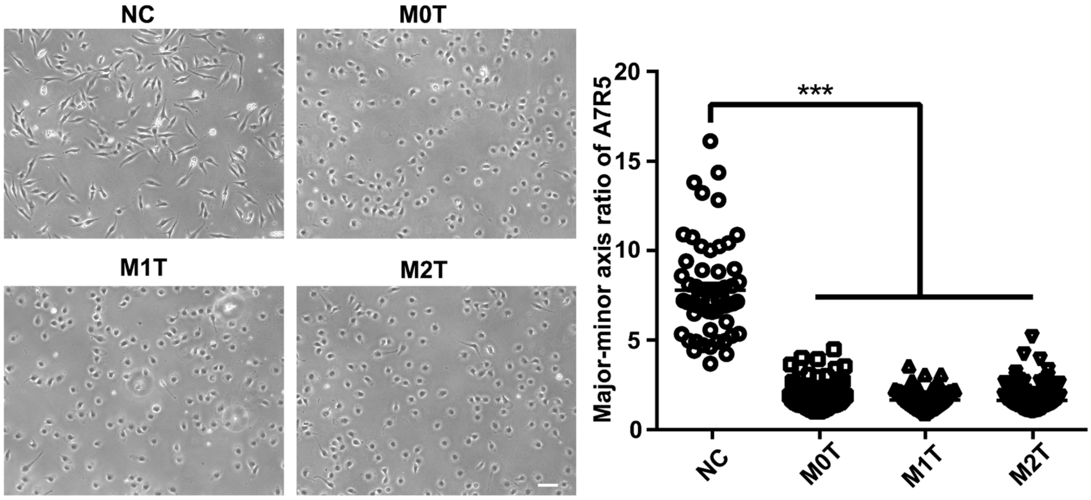

Figure S4. The axis ratio of VSMCs decreased under the stimulation of macrophages. VSMCs were co-cultured with macrophages through Transwell co-culture system for 24 h. Scale bar = 10 μm. ***P < 0.001 versus the control group at the corresponding times and area; one-way, repeated-measures ANOVA.

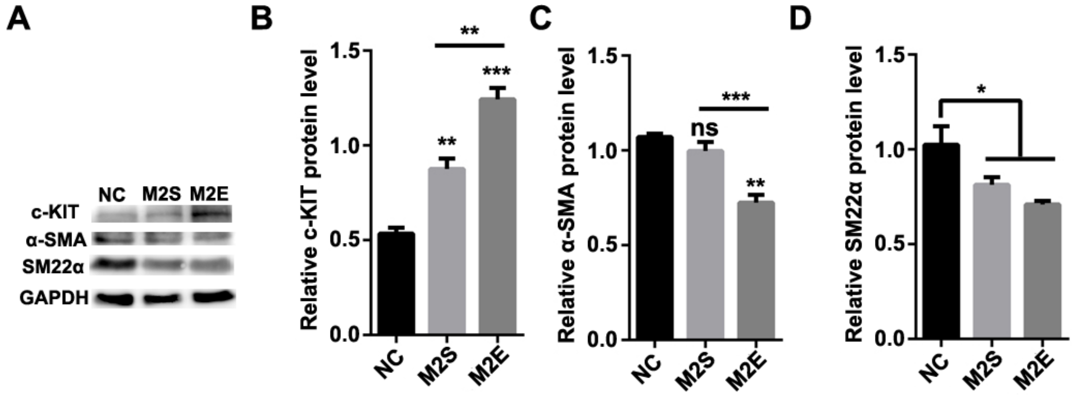

Figure S5. M2 macrophages promoted VSMC dedifferentiation by upregulating c-KIT and downregulating SM22α and α-SMA. A. Representative immunoblots for SMC differentiation markers (SM22α and α-SMA) and stem cell markers (c-KIT) in lysates from quiescent cells grown in DMEM media free-FBS for 24 h. B, C and D. The levels of c-KIT, SM22α and α-SMA were determined using specific antisera against these antigens. Data are representative of blots with similar results. Equal loading was confirmed by Ponceau S staining of the membranes and by measuring the constitutive GAPDH gene. Error bars represent means ± SEM. P values indicate the

significance of differences of treatments versus the relevant control. ***P < 0.001 by one-way, repeated-measure ANOVA.

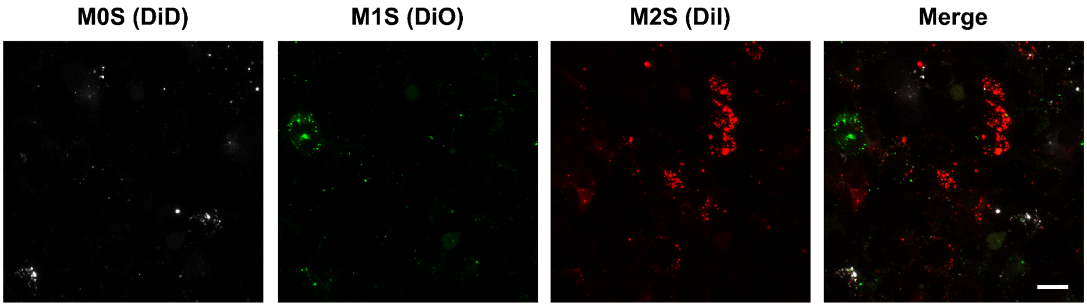

Figure S6. The absorption of fluorescent labeled EVs from M0, M1 and M2 macrophage supernatant by the VSMCs for 24 h. Scale bar = 10 µm.

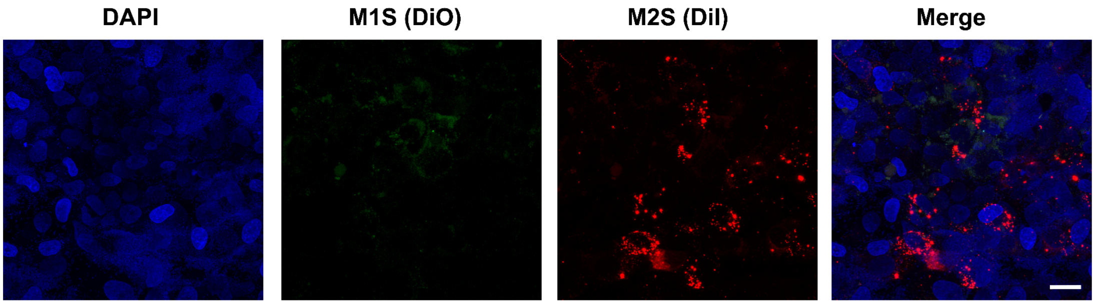

Figure S7. The absorption of fluorescent labeled EVs from M1 and M2 macrophage supernatant by the VSMCs for 24 h. Scale bar = 10 µm.

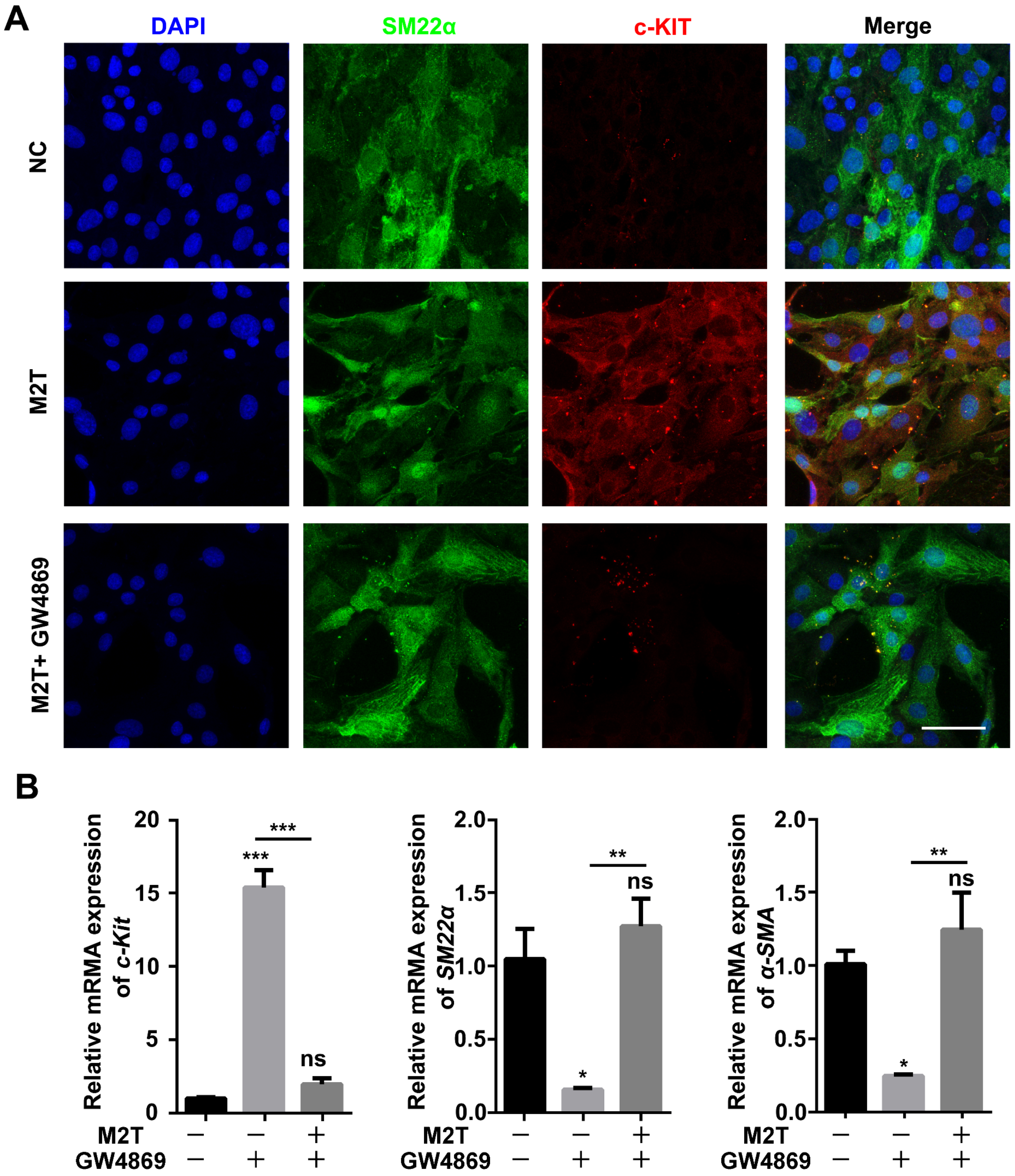

Figure S8. c-KIT expression in the VSMCs was affected by M2 macrophage derived exosomes. (A) Co-immunostaining for SM22α (green) and c-KIT (red) in RASMCs 24 h after culture in the presence of M2E and/or GW4869 (10 μM). Nuclei were stained with DAPI (blue). Scale bar = 10 μm. (B) Quantification of relative mRNA expression of *SM22α*, *α-SMA* and *c-Kit* in NC, M2T and M2T+GW4869. Error bars are Mean ± SEM. *P < 0.05, **P < 0.01, ***P < 0.001 (one-way, repeated-measure ANOVA).

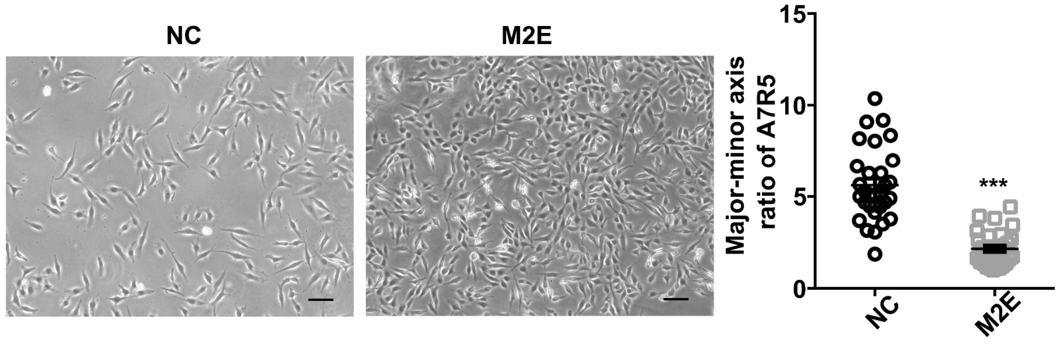

Figure S9. The axis ratio of VSMCs decreased under the stimulation of M2 macrophages derived exosomes. VSMCs were co-cultured with M2 macrophage-derived exosomes for 24 h. Scale bar = 100 µm. ***P < 0.001 versus the control group at the corresponding times and area (Student's unpaired, two-sided t-test).

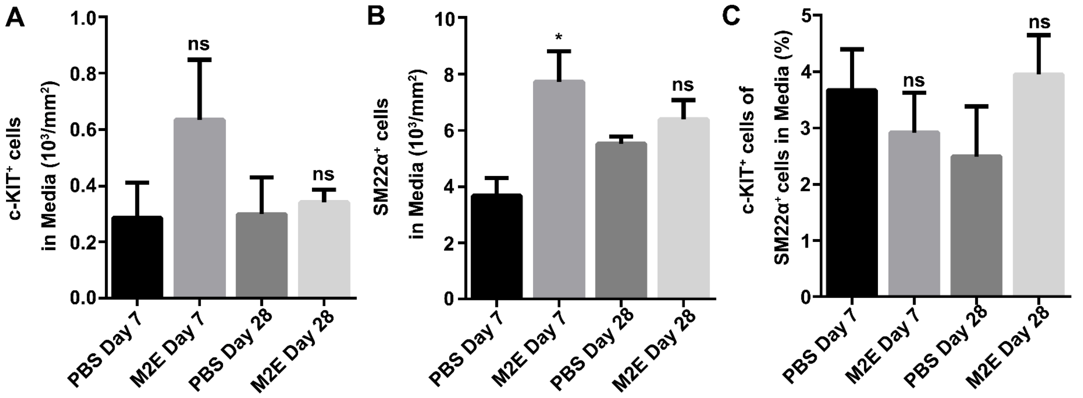

Figure S10. Analysis of positive cells counted in the media after the abdominal aortic stent implantation with M2Es or PBS treatment at 7 days and 28 days post-stenting. (A) The number of c-KIT$^+$ cells were counted in the media. (B) The number of SM22α$^+$ cells were counted in the media. (C) Percentage of c-KIT and SM22α double-positive cells of SM22α$^+$ cells in the media. N = 7 for each group. *P < 0.05 versus the PBS-treated group at the corresponding times and area (one-way, repeated-measures ANOVA).

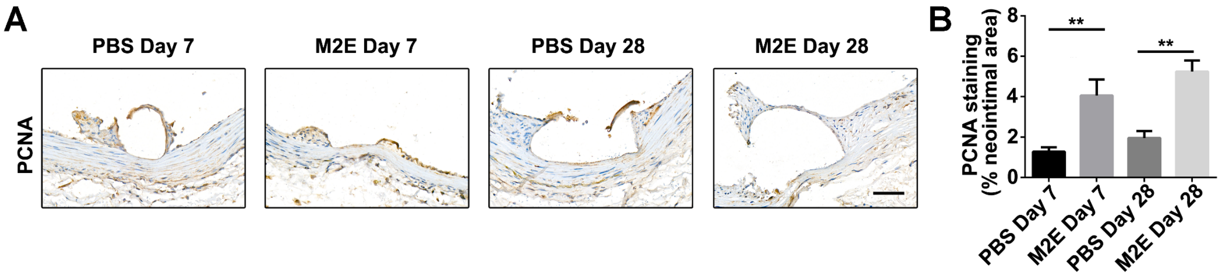

Figure S11. Cellular proliferation analysis of the stented rats' lesions. The cellular composition of the neointimal lesions was quantified in PBS-treated and M2E-treated rats at 7 days and 28 days post-stenting, showing quantification of: (A and B) percentage of cells staining positive for proliferating cell nuclear antigen (PCNA). Scale bar = 50 μm. N = 7 for each group. **p < 0.01 versus PBS-treated groups (one-way, repeated-measures ANOVA).

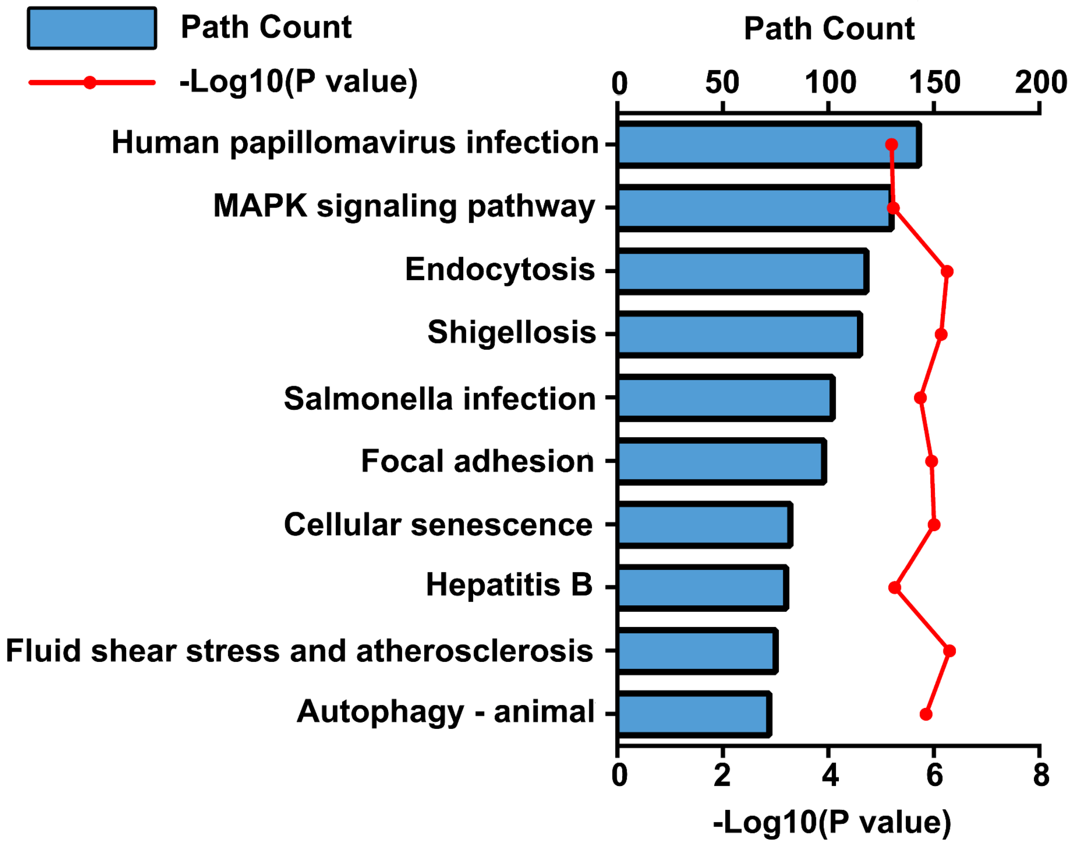

Figure S12. Kyoto Encyclopedia of Genes and Genomes pathway analysis of the identified target genes.

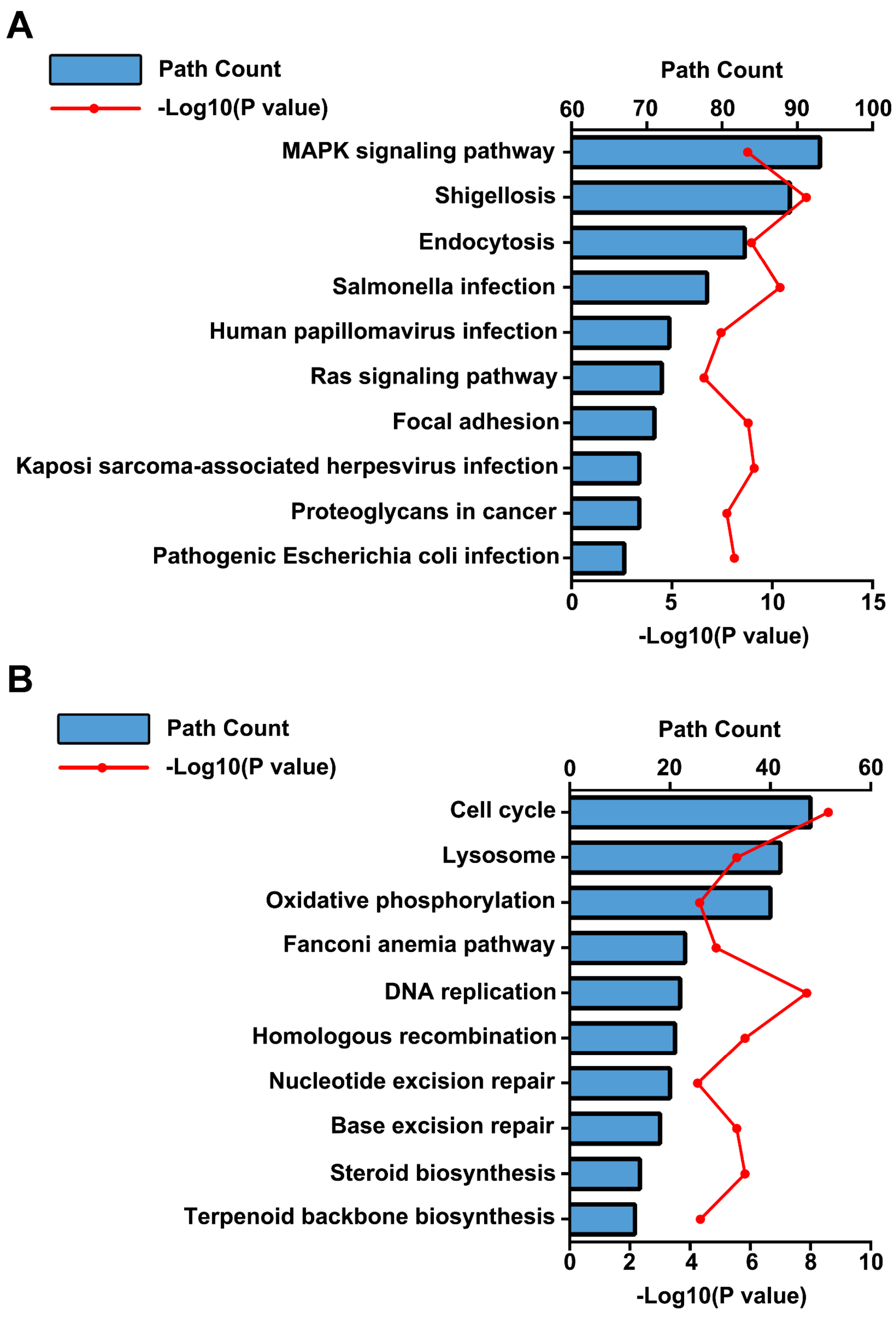

Figure S13. Kyoto Encyclopedia of Genes and Genomes pathway analysis of up (A) /down (B) regulation of key pathways in VSMCs.

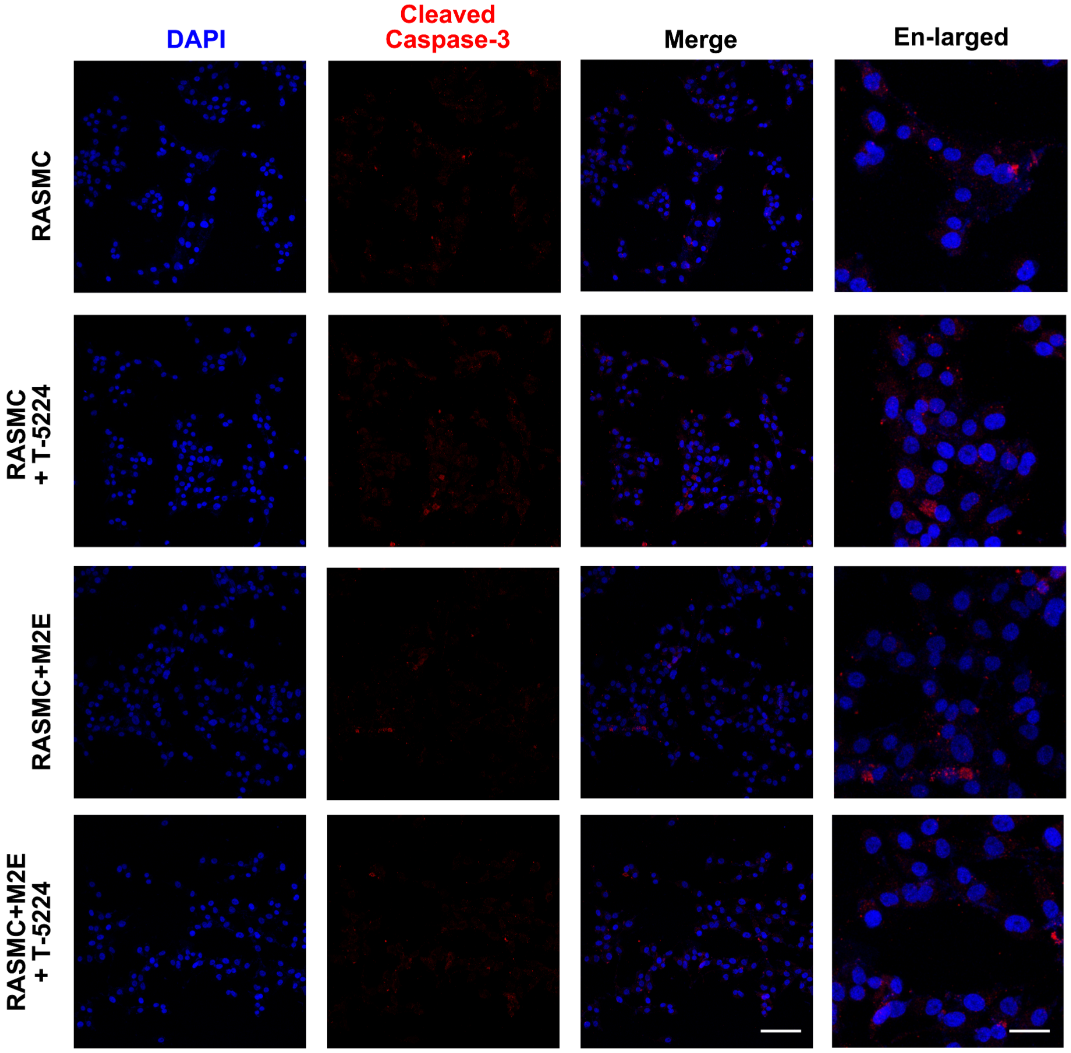

Figure S14. The inhibition of AP1 did not affect the apoptosis of RASMCs. Representative images of RASMCs labeled with cleaved caspase-3 (red) 24 h after culture in the presence of M2Es and/or T-5224 (10 μM). Nuclei were stained with DAPI (blue). Scale bar = 50 μm, enlarged view, scale bar = 10 μm.

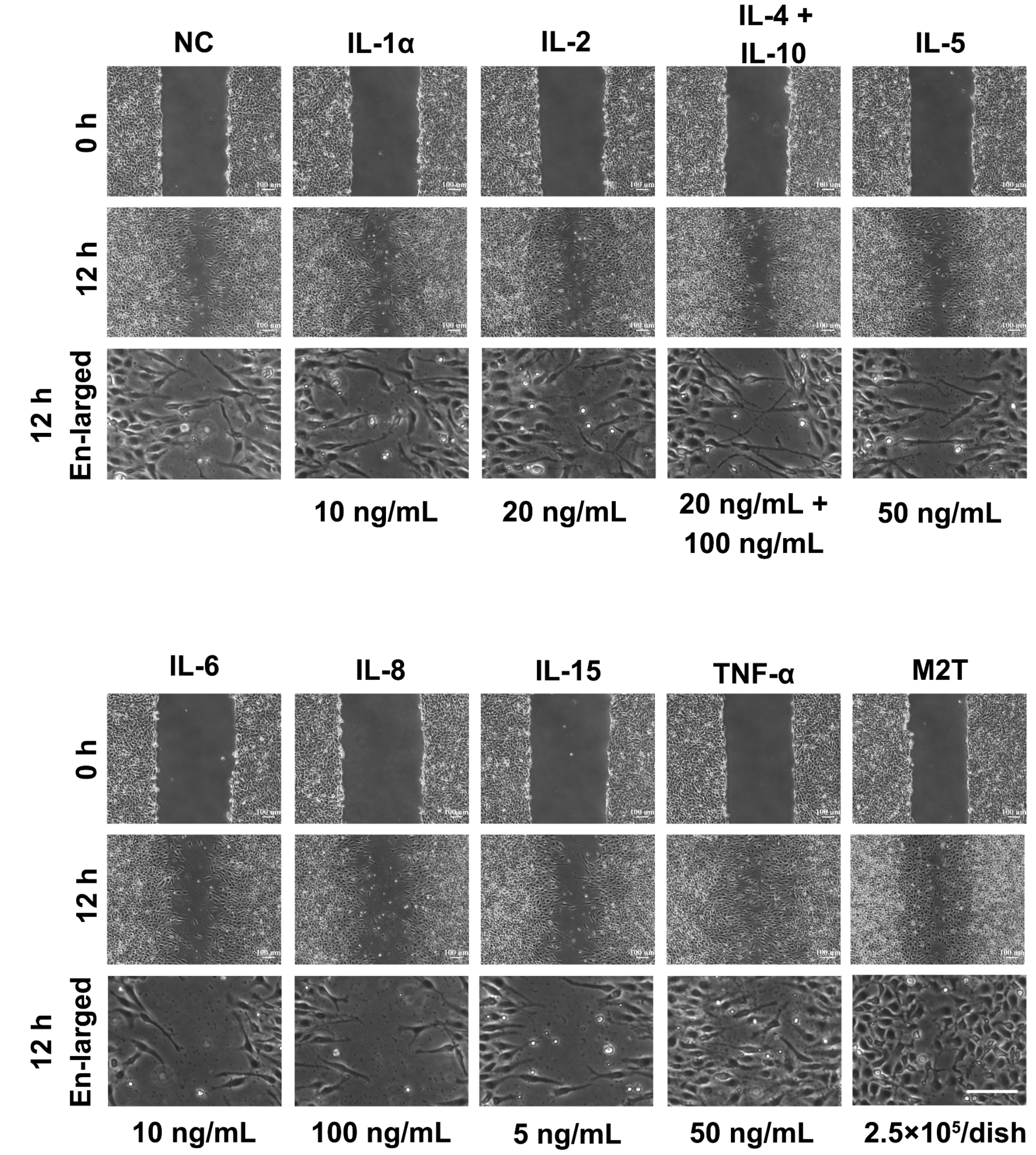

Figure S15. Quiescent scratched VSMCs were treated with IL-1α (10 ng/mL), IL-2 (20 ng/mL), IL-4+IL-10 (20 ng/mL + 100 ng/mL), IL-5 (50 ng/mL), IL-6 (10 ng/mL), IL-8 (100 ng/mL), IL-15 (5 ng/mL) and TNF-α (50 ng/mL) in FBS-free medium for 12 h. VSMCs co-cultured with M2 macrophages served as a positive control. Phase-contrast microscopy images showing the morphologic changes and migratory capacity of VSMCs under different treatment. Scale bar = 100 µm, En-larged: Scale bar = 50 µm.

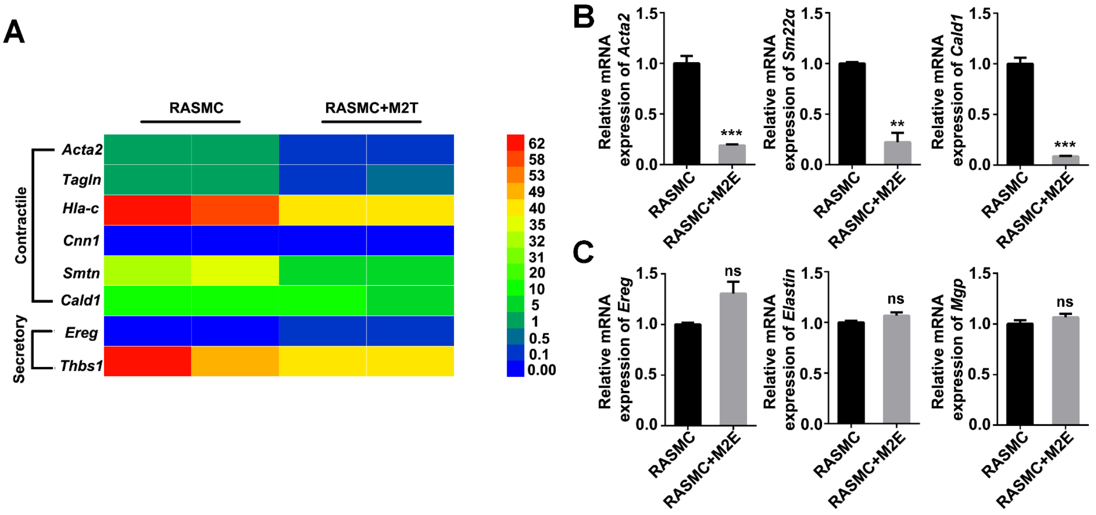

Figure S16. Verification of typical phenotype changes in the VSMCs. (A) Heatmap displaying genes that were differentially expressed between RASMCs and RASMCs co-cultured with M2 macrophages. (B and C) qRT-PCR showed that the expression of secretory or contractile state genes in the RASMCs and M2Es-treated RASMCs. Dates represent means ± SEM. $^{**}p < 0.01$, $^{***}p < 0.001$ (Student's unpaired, two-sided t-test);
